# Quantum spin Hall effect in III-V semiconductors at elevated temperatures: Advancing topological electronics

**DOI:** 10.1126/sciadv.adz2408

**Published:** 2025-10-24

**Authors:** Manuel Meyer, Jonas Baumbach, Sergey Krishtopenko, Adriana Wolf, Monika Emmerling, Sebastian Schmid, Martin Kamp, Benoit Jouault, Jean-Baptiste Rodriguez, Eric Tournie, Tobias Müller, Ronny Thomale, Gerald Bastard, Frederic Teppe, Fabian Hartmann, Sven Höfling

**Affiliations:** ^1^ Julius-Maximilians-Universität Würzburg, Physikalisches Institut and Würzburg-Dresden Cluster of Excellence ct.qmat, Lehrstuhl für Technische Physik, Am Hubland, 97074 Würzburg, Germany.; ^2^ Laboratoire Charles Coulomb (L2C), UMR 5221 CNRS-Université de Montpellier, Montpellier, France.; ^3^ Physikalisches Institut and Röntgen Center for Complex Material Systems, 97074 Würzburg, Germany.; ^4^ IES, Université de Montpellier, CNRS, F-34000 Montpellier, France.; ^5^ Institut Universitaire de France, F-75005 Paris, France.; ^6^ Institut für Theoretische Physik und Astrophysik, Universität Würzburg, Würzburg, Germany.; ^7^ Department of Physics, University of Zurich, Winterthurerstrasse 190, 8057 Zurich, Switzerland.; ^8^ Physics Department, École Normale Supérieure, PSL 24 rue Lhomond, 75005 Paris, France.

## Abstract

The quantum spin Hall effect (QSHE), a hallmark of topological insulators, enables dissipationless, spin-polarized edge transport and has been predicted in various two-dimensional materials. However, challenges such as limited scalability, low-temperature operation, and the lack of robust electronic transport have hindered practical implementations. Here, we demonstrate the QSHE in an InAs/GaInSb/InAs trilayer quantum well structure operating at elevated temperatures. This platform meets key criteria for device integration, including scalability, reproducibility, and tunability via electric field. When the Fermi level is positioned within the energy gap, we observe quantized resistance values independent of device length and in both local and nonlocal measurement configurations, confirming the QSHE. Helical edge transport remains stable up to *T* = 60 kelvin, with further potential for higher-temperature operation. Our findings establish the InAs/GaInSb system as a promising candidate for integration into next-generation devices harnessing topological functionalities, advancing the development of topological electronics.

## Teaser

Engineered materials sustain quantum spin Hall edge transport at elevated temperatures, advancing next-generation electronics.

## INTRODUCTION

In recent years, topological insulators (TIs) exhibiting the quantum spin Hall effect (QSHE) have attracted considerable attention due to their fascinating electronic properties, characterized by an insulating bulk and gapless helical edge states (*1*–*4*). These properties make them promising for fundamental research as well as for potential device applications using dissipationless and spin-polarized transport of electrons (*5*). After the theoretical prediction of the QSHE in graphene (*1*) and later in inverted HgTe/CdTe quantum wells (QWs) (*6*), its experimental verification was followed by observing the expected quantized resistance within the bandgap (*7*). The nature of the edge states was further confirmed by measurements in nonlocal geometries (*8*, *9*). In addition, various other material systems have been proposed to host the QSHE associated with the topological band structure (*10*–*16*). However, to date, any unambiguous observation of the conductance quantization resulting from the QSHE in other systems has only been observed in monolayer (ML) WTe_2_ (*17*) and InAs/GaSb bilayer quantum wells (BQWs) (*18*, *19*). While in WTe_2_ the QSHE was even robust up to *T* = 100 K, the complex fabrication of these two-dimensional (2D) materials hampers the scalability required for widespread technological development. In HgTe QWs, the limiting factor is the maximal operation temperature, i.e., the QSHE was only observed up to 15 K (*20*). Although bandgaps up to 55 meV in HgTe QWs in the TI phase are anticipated via strain engineering (*21*), the pronounced temperature dependence of the band ordering and phase transition to a normal insulating (NI) phase ultimately restricts the QSHE to low temperatures, thereby limiting the use of HgTe QWs in topological electronics applications (*22*–*24*). This leaves the InAs/GaSb material system as a promising candidate in terms of scalability, reproducibility, and electrical tunability of helical edge channel transport at elevated temperatures. It benefits from a rather temperature-independent band ordering indicated through independent transport and terahertz spectroscopy techniques (*25*–*28*) and the maturity of the growth and processing of III-V semiconductors, including the compatibility with Si-based chips (*29*). Further device functionality in this material system arises from the inherent property of the spatially separated electron and hole gases in the InAs and GaSb layers, respectively. This allows for tunability between trivial and topological band orderings, enabling the phase transition between the TI and NI phases imposed by external electric fields (*30*). While such a phase transition was also observed in Na_3_Bi, which benefits from large TI and NI gaps (*31*, *32*), the required electrical fields are difficult to achieve with conventional gates, and the conductance quantization was not observed. However, the inherently small bandgap of a few meV in InAs/GaSb BQWs (*30*, *33*, *34*) limited the maximum observation temperature of the QSHE to 4 K (*19*). One possibility to increase the bandgap in inverted InAs/GaSb-based heterostructures is to replace the GaSb layers with Ga_1−*x*_In*_x_*Sb alloys resulting in a higher overlap of the electron and hole wave functions at the InAs/GaInSb interfaces. This replacement substantially enhances the inverted bandgap as it was first found for strained InAs/GaInSb superlattices (*35*). This idea was later applied to InAs/Ga_1−*x*_In*_x_*Sb BQWs (*36*–*38*), in which the bandgap could be increased to 25 meV for *x* = 40. Alternatively, the bandgap in InAs/GaSb-based QWs can be increased several times by eliminating the structure inversion asymmetry (SIA) inherent in the BQWs by adding an additional InAs layer (*25*). Thus, by combining these two ideas, the bandgap in inverted InAs/GaInSb/InAs trilayer quantum wells (TQWs) is predicted to be as high as 50 to 60 meV for realistic TQW parameters (*25*). Recent magneto-transport studies on gated Hall bars fabricated from InAs/Ga_0.65_In_0.35_Sb/InAs TQWs revealed bandgap values of 45 meV (*25*, *39*). These relatively large bandgap values should allow excluding parasitic contributions into the QSHE, such as residual bulk conductivity and trivial edge states (*19*, *36*, *40*–*42*) and should enable operations at elevated temperatures.

In this work, we report on the observation of a length-independent quantized resistance value up to elevated temperatures in TIs based on InAs/GaInSb/InAs TQWs with a moderate bandgap. The absence of length-dependent resistances within the bandgap in both local and nonlocal measurement geometries provide compelling evidence of QSHE, suggesting transport exclusively through helical edge states. For devices with deviating resistances in the gap, the gate-training technique is used to obtain a quantized resistance value. Last, we investigate the robustness of the QSHE against temperature. We find that the resistance in the bandgap remains stable up to *T* = 60 K, with the potential to observe the QSHE at even higher temperatures.

## RESULTS

### Length-independent conductance quantization

**Fig. 1.**
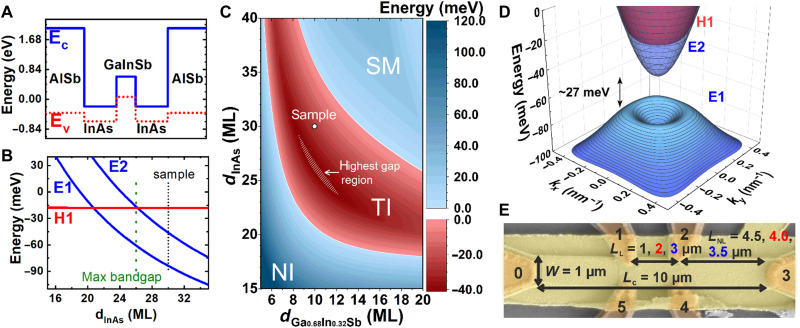
Details about the sample. (**A**) Band-edge diagram for symmetric InAs/Ga_0.68_In_0.32_Sb TQWs grown on (001) AlSb buffer. (**B**) Subband energies at zero quasimomentum *k* in the TQW as a function of InAs layer thickness d_InAs_. Blue and red curves correspond to the electron-like (E1 and E2) and hole-like (H1) states, respectively. The thickness of the GaInSb layer d_GaInSb_ equals 10 monolayers (MLs). (**C**) Color map diagram for symmetric InAs/Ga_0.68_In_0.32_Sb TQWs grown on (001) AlSb buffer as a function of *d*_InAs_ and *d*_GaInSb_ with an NI, TI, and SM region. The highest bandgap region in the TI regime and the sample studied in this manuscript are marked as a white hatched region and with an open symbol, respectively. For the SM region, the bandgap is zero, while the energy here marks the overlap between the maximum of the valence band and the minimum of conduction band. (**D**) 3D plot of the band structure of the investigated sample with the *x* and *y* axes being the [100] and [010] crystallographic directions, respectively. (**E**) Colored scanning electron microscopy image of a Hall bar device. The contacts are labeled from 0 to 5 with a length of *L*_C_ = 0.2 μm and the total length and width of the Hall bar are *L* = 10 μm and *W* = 1 μm, respectively. For the upper and lower contacts, different contact separation lengths *L*_L_ = 1, 2, and 3 μm and *L*_NL_ = 4.5, 4.0, and 3.5 μm were chosen to perform length-dependent measurements in local and nonlocal geometries.

**Fig. 2.**
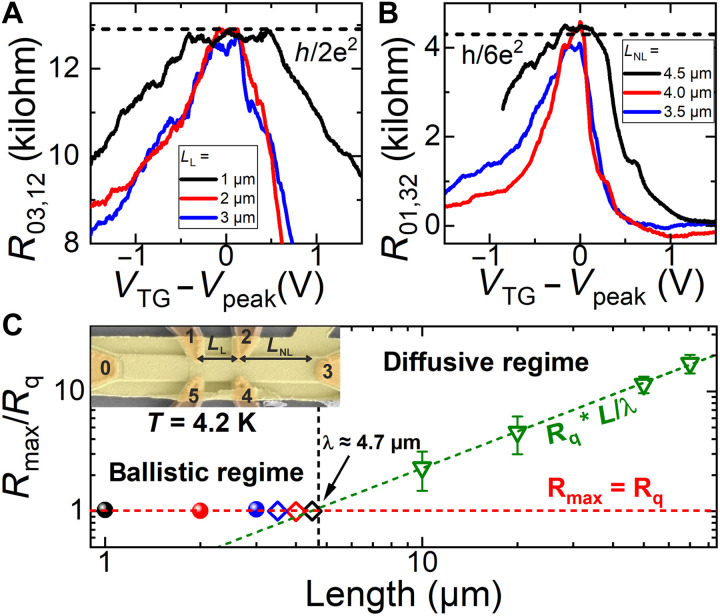
Length-independent conductance quantization at 4.2 K. Local resistance *R*_03,12_ and nonlocal resistance *R*_01,32_ in (**A**) and (**B**), respectively, as a function of *V*_TG_-*V*_peak_ for gated devices with different lengths *L*_L_ and *L*_NL_. The resistance in the bandgap is length independent and quantized to the expected value of *h*/2*e*^2^ and *h*/6*e*^2^, for local and nonlocal configurations, respectively. (**C**) Maximum edge resistance values normalized to the expected quantized value *R*_q_ for Hall bar devices of different lengths. Large-scaled Hall bars (green triangles) show a length dependence for the gap resistance in the diffusive regime in comparison to the Hall bars with *L*_L_ = 1, 2, and 3 μm in the ballistic regime.

**Fig. 3.**
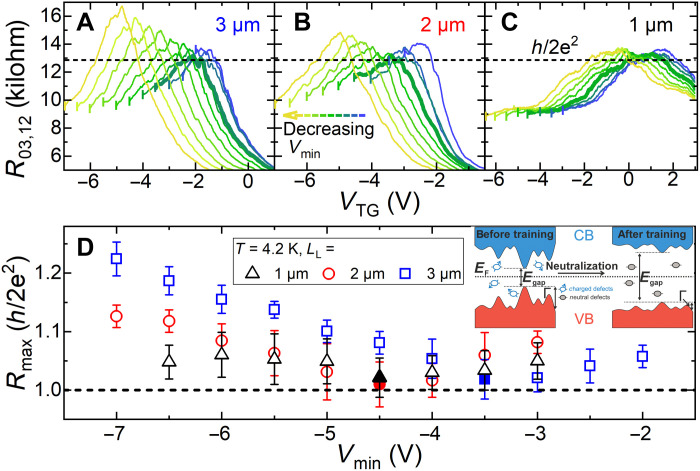
Improving the quantization via the gate-training technique. Gate-training technique was applied to three devices with *L*_L_ = 3, 2 and 1 μm in (**A**), (**B**), and (**C**), respectively. By sweeping the top gate to an optimized minimum gate voltage *V*_min_ and back through the bandgap, the resistance in the gap is approaching the quantized value of *h*/2*e*^2^ (curves marked in bold). For nonoptimized gate voltage sweeps, the coherence length of the edge channels is shorter than the contact separation lengths and hence *R* > *h*/2*e*^2^. (**D**) shows the evolution of the maximum resistance *R*_max_ versus *V*_min_ with the best resistance values close to *h*/2*e*^2^ for optimal gate training shown with filled symbols. The inset qualitatively illustrates the improvement through the gate training (left, before training; right, after training) on the potential landscape and the neutralization of charged defects in the vicinity of the edge channels.

**Fig. 4.**
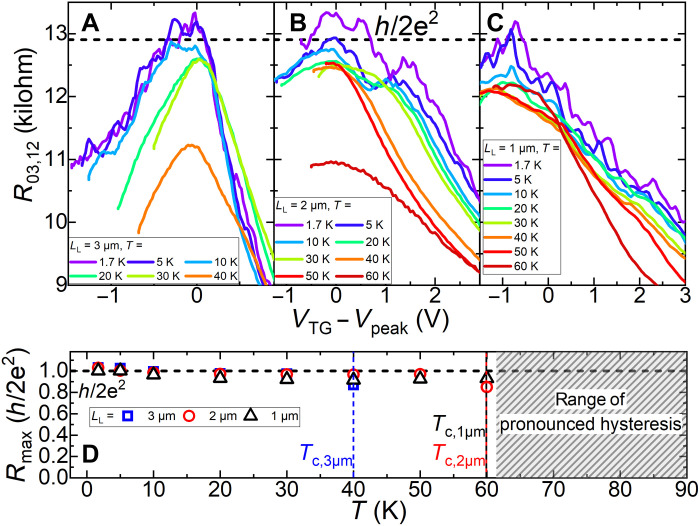
Robust conductance quantization up to *T* = 60 K. Temperature dependence of the quantized resistance value in the bandgap for *L*_L_ = 3, 2 and 1 μm in (**A**), (**B**), and (**C**), respectively. The resistance maximum values are summarized in (**D**). For all three devices, a constant *R*_max_ at *h*/2*e*^2^ can be observed with a maximum temperature of *T* = 60 K for the 1- and 2-μm Hall bar devices. At larger temperatures, the gate-training range exceeds the accessible voltage range, at which the gate operates stably (grayed region).

Figure 1A schematically shows the band-edge diagram for InAs/Ga_0.68_In_0.32_Sb/InAs TQWs confined by outer AlSb barriers grown on a (001) AlSb buffer layer. The broken-gap alignment at the InAs/(Ga,In)Sb interface leads to the possibility of the band inversion in InAs/(Ga,In)Sb-based QWs, when the first electron-like (E1) subband at zero wave vector *k* lies below the first hole-like (H1) subband, which is realized at certain thicknesses of InAs and (Ga,In)Sb layers. The interaction between the E1 and H1 subbands at a nonzero wave vector opens a hybridization gap in the BQW, resulting in 2D time-reversal invariant TI state (*15*). In contrast, the presence of the second InAs layer in the TQW eliminates the SIA in the growth direction and the bandgap under inversion between the E1 and H1 subbands opens mainly due to the confinement effect at zero *k*, similar to the case of HgTe QWs (*6*, *7*). As the band structure calculations show, the inverted bandgap in such QW geometry greatly exceeds the bandgap values in InAs/GaInSb BQWs with the same layer materials (*25*). Figure 1B presents the position of electron-like and hole-like subbands at *k* = 0 in the TQW as a function of InAs-layer thickness *d*_InAs_ for GaInSb-layer thickness of *d*_GaInSb_ = 10 MLs, where 1 ML corresponds to the half of a lattice constant in the bulk material. As the wave functions in electron-like subbands are localized in the InAs layers, while they are mostly localized in the GaInSb layers in the hole-like subbands, the symmetric TQW can be interpreted as a “double QW for electrons” with a GaInSb middle barrier, which likewise takes on the role of a “QW for holes” (see Fig. 1A). In this case, the E1 and E2 levels can be interpreted as even-odd state splitting arising from the tunnel-coupled “QWs for electrons.” If InAs layers are thin, the E1 subband lies above the hole-like H1 subband, and the TQW has a trivial band ordering, representing a NI state. Increasing *d*_InAs_ induces the mutual inversion between E1 and H1 subbands, resulting in inverted band structure of the TQW. The bandgap in the inverted TQWs has a nonmonotonic dependence on the InAs layer thickness. As *d*_InAs_ increases, the gap first becomes indirect, reaching its maximum value when E2 and H1 subbands become close to each other (see Fig. 1B), and then closes due to indirect overlap of the conduction (E2) and valence (E1) bands at a semimetal (SM) state (*25*). A complete phase diagram as a function of *d*_InAs_ and *d*_GaInSb_ for symmetric InAs/Ga_0.68_In_0.32_Sb/InAs TQW grown on (001) AlSb buffer is depicted in Fig. 1C. The white hatched region represents the region with the largest TI bandgaps above 40 meV, which can be achieved for a given indium content of 32% in the GaInSb alloy. The TQW sample used for the observation of the QSHE consists of two InAs layers with a thickness of *d*_InAs_ = 30 ML (≈9.1 nm) and one Ga_0.68_In_0.32_Sb layer with a thickness of *d*_GaInSb_ = 10 ML (≈3.1 nm). The chosen layer thicknesses result in a topological band structure with a moderate inverted bandgap energy of approximately *E*_gap_ = 27 meV as shown in the 3D dispersion in Fig. 1D. Here, the *x* and *y* axes represent the [100] and [010] crystallographic directions, respectively. To observe conductance quantization, the lengths between all the contacts of the investigated devices must be smaller than the phase coherence length λ, which refers to a length scale at which dissipationless edge transport breaks down and counterpropagating spin-up and spin-down channels equilibrate. This results in a deviation of the resistance from the expected quantized value (*43*). Therefore, Hall bars of the investigated InAs/GaInSb/InAs TQW were fabricated, such that contact separation lengths are equal or smaller than what was typically observed in this system (*18*, *36*, *39*). Details about the sample and band structure calculations can be found in Materials and Methods and in fig. S1. Temperature-dependent measurements to confirm the bandgap can be found in fig. S2. Figure 1E shows a typical scanning electron microscopy image of such a Hall bar device. The total length of the devices is always *L* = 10 μm and the width *W* = 1 μm. All six contacts are symmetrically arranged with a length of *L*_C_ = 0.2 μm and are labeled from 0 to 5. For the contact separation length *L*_L_ (from middle to middle) between the upper (1 and 2) and lower (4 and 5) contacts, variations of *L*_L_ = 1, 2, and 3 μm were chosen. This results in a separation *L*_NL_ between the outer contacts of *L*_NL_ = 4.5, 4.0, and 3.5 μm, so *L* = *L*_L_ + 2*L*_NL_ = 10 μm. On the basis of the literature (*18*, *36*, *39*), these lengths should be equal or below the typical phase coherence lengths of TIs based on the InAs/(Ga,In)Sb system. All values for *L*_L_ and *L*_NL_ are colored coded in black, red, and blue. In total, about 20 devices have been studied, where no top-gate leakage current was observed for *V*_TG_ = +10 to −10 V. Three additional devices were used for each series of measurements reported in Figs. 2 to 4. Therefore, the results in this manuscript are representative for all the devices studied. The results are also reproducible between different thermal cycles. To observe conductance quantization and prove the QSHE in InAs/GaInSb/InAs TQWs, measurements in both local and nonlocal geometries for different contact separation lengths were performed. If not stated otherwise, all measurements are performed in the dark and at *T* = 4.2 K. The experiments were always conducted in four-probe configuration, which directly eliminates contact resistance. For the typical local configuration shown in Fig. 2A, the top-gate voltage (*V*_TG_) is swept through the gap, and the resistance *R*_03,12_ (a 100 nA current is applied from 0 to 3, and the voltage is measured between 1 and 2) is recorded. In the following, all the curves were shifted horizontally to match the position of the peak resistance. For three different lengths *L*_L_ = 1, 2, and 3 μm (in black, red, and blue, respectively), the peak resistance value coincides with the expected quantized value of h/2e^2^ (see fig. S3 for a calculation of the expected quantized values). This length-independent resistance in the gap proves the QSHE and ballistic transport through the 1D edge channels as it is expected if the length of the edge channels is smaller than λ (*7*). The voltage values of the peak are: *V*_peak_ = −2.19,−1.47, and −1.96 V for the 1-, 2-, and 3-μm device, respectively. Furthermore, broad resistance plateaus with fluctuations can be observed, as usual for the QSHE at cryogenic temperatures (*7*, *44*, *45*). The broader peak and larger resistance for the electron and hole regime for the 1-μm device can be explained by a weaker gate efficiency. This is indicated by the weaker dependence of resistance on the top-gate voltage. Therefore, the effective range, where the Fermi energy is tuned in this device, is also smaller than for the other two devices. Transport measurements in the nonlocal configuration (current applied from probes 0 to 1, voltage measured between probes 2 and 3) for different lengths of *L*_NL_ = 4.5, 4.0, and 3.5 μm are presented in Fig. 2B. The voltage values of the peaks for the nonlocal measurements are: *V*_peak_ = −2.15, −2.45, and − 2.21 V for the 1-, 2-, and 3-μm device, respectively. The different peak positions for local and nonlocal configurations for the same devices result from different gate-voltage ranges, which were used for the experiments. For the same parameters, the peak is always found at the same gate-voltage position irrespective of local or nonlocal geometries, as can be seen in fig. S4. For these devices, a length-independent nonlocal resistance *R*_01,32_ was observed, which was quantized to the expected value of h/6e^2^ (*41*). Additional measurement configurations showing quantization can be found in fig. S5. This further confirms the observation of the QSHE and that the transport is provided only at the edges through the helical edge channels in the gap similar to what was previously observed in HgTe/CdTe QWs (*8*) and InAs/(Ga,In)Sb BQWs (*19*, *36*).

Various devices have been tested to obtain definite results for the length independence of the resistance. The average values of the maximum resistance *R*_max_ of all the Hall bars investigated were normalized to the expected quantized value *R*_q_ and are summarized in Fig. 2C. *R*_max_/*R*_q_ = 1 indicates a perfect quantization. The extracted resistances for the devices in both the local (balls) and nonlocal (diamonds) measurement geometries are shown. Up to a length of *L* = 4.5 μm, the average values are in good agreement with the expected value, which already indicates λ ≥ 4.5 μm. In comparison, the results obtained on large-scaled Hall bars with *W* = 20 μm and *L*_L_ = 10, 20, 50, and 70 μm (green triangles) show a clear length dependence of the edge resistance maximum as expected. These results on the edge resistance for the large-scale devices have been obtained by a detailed analysis to separate edge and bulk contributions (*46*). This demonstrates the transition from the length-dependent diffusive regime to the length-independent ballistic regime as the resistance saturates at the quantized value in comparison to what was observed for trivial InAs/GaSb BQWs (*42*). The quantization in the length-independent regime indicates an insulating bulk and no substantial parasitic contribution to the transport through helical edge channels, as is further confirmed in fig. S6. Furthermore, the intersection of the linear fit with *R*_max_/*R*_q_ = 1 allows us to extract the phase coherence length to λ ≈ 4.7 μm. A detailed analysis of this extraction can be found in figs. S7 and S8. As all our contact separation lengths in the microscopic devices are smaller than 4.5 μm, this qualitatively confirms the fact that the quantized resistance values are not the result of an arbitrary coincidence but due to the observation of QSHE. The QSHE is further confirmed by a demonstration of breaking of time-reversal symmetry in figs. S9 to S11.

### Approaching quantization through gate training

As previously shown for HgTe QWs (*47*), the phase coherence length of the helical edge states can be increased using the gate-training technique, which is sweeping the gate voltage to minimum values *V*_min_ and then back through the bandgap region. The backscattering and decrease of λ is associated to the spin of charged defects in the vicinity of the helical edge states (*46*, *48*, *49*). In TIs based on InAs/(Ga,In)Sb, such charged defects may also cause backscattering like in HgTe QWs (*50*). The charged defects also lead to a decrease in the quantum scattering time τ_q_, which results in pronounced fluctuations in the potential landscape that can be quantified as the quantum level broadening Γ = ℏ/2τ_q_. This effectively decreases the bandgap. By applying the gate-training technique, these charged defects in the vicinity of the TQW are neutralized (*50*). This neutralization improves the quantum scattering time τ_q_ and Γ, and the backscattering is decreased. Therefore, the resistance in the gap approaches quantization for an optimized *V*_min_ (*47*, *50*). The gate-training technique was used on three devices with *L*_L_ = 3, 2, and 1 μm from the same TQW, where the resistance deviated from the expected quantized value, as shown in Fig. 3, A to C. The top gate was swept to different *V*_min_ = −2 to −7 V (indicated by a small vertical bar on the left side of all measured curves in Fig. 3, A to C) and back through the gap. For the up-sweeps (when sweeping from *V*_min_ back to positive voltages), the curves are optimized, and the resistances are closest to the quantized value of *h*/2*e*^2^ for *V*_min_ = −4.5 V (for *L*_L_ = 1 and 2 μm) and *V*_min_ = −3.5 V (for *L*_L_ = 3 μm). These curves are marked in bold. By extracting *R*_max_ and plotting it as a function of *V*_min_ in Fig. 3D, this improvement can be observed more clearly. The error bars for *R*_max_ are determined by the resistance fluctuations in the vicinity of the maximum peak values (*R*_max_ ± 5%). The inset in Fig. 3D qualitatively illustrates the gate training effect (left, before training; right, after training) on the potential landscape and the neutralization of charged defects in the vicinity of the edge channels. The device with *L*_L_ = 3 μm exhibits the largest improvement of the quantization by gate training, whereas only a minimal enhancement was achieved for the device with *L*_L_ = 1 μm. The lowest values were: *R*_max,3μm_ = (1.018 ± 0.032), *R*_max,2μm_ = (1.009 ± 0.038), and *R*_max,1μm_ = (1.022 ± 0.017), in units of *h*/2*e*^2^. The same minimum resistance value is approached for all devices, which is also the expected quantized resistance value for this configuration. This provides further evidence for the length-independent resistance in the gap and the QSHE, while also highlighting the advantage of gate training in enhancing the reproducibility of quantization.

### QSHE at elevated temperatures

As the band ordering in the InAs/(Ga,In)Sb material system is rather temperature insensitive (*26*, *27*), we investigated the robustness of the helical edge channels for different temperatures. Figure 4 (A to C) represents the local resistances *R*_03,12_ for Hall bar devices with *L*_L_ = 3, 2, and 1 μm at increasing temperatures. The gate-training technique has been used at each temperature. *R*_max_ is presented as a function of temperature in Fig. 4D. *R*_max_ remained rather constant at *R*_*m*ax_ = *h*/2*e*^2^ until they reached their respective threshold temperatures *T*_c_. These are: *T*_c,3μm_ = 40 K, *T*_c,2μm_ = 60 K, and *T*_c,1μm_ = 60 K, while it seems to be the most robust for *L*_L_ = 1 μm. This robustness of the helical edge channels for smaller devices is further supported by a model of edge resistance with additional backscattering in fig. S12. The onset temperature of quantized conductance was extracted as in (*17*) with a maximal deviation of up to 15%. The observation of conductance quantization up to 40 K can be attributed to the increase in bandgap energy, and subsequent reduction of bulk conductivity. This maximum operation temperature could be reproducibly achieved in the devices investigated. However, the achieved quantization up to 60 K, as presented for the 1- and 2-μm devices, stems from device-to-device variations in the residual bulk conductivity and subsequent larger bulk resistance as obtained from the large-scale Hall bar data (see fig. S6). These variations could be due to growth or processing, which requires further investigations. With improvements in sample growth and fabrication, these variations can be improved in the future. The microscopic devices further exhibit a low mobility (see fig. S13) leading to an overall larger bulk resistivity. We also emphasize that since the resistance at the maximum operation temperature remains close to the quantized value for all devices, the bulk resistance must still exceed the helical edge resistance, indicating that transport is dominated by the edges.

In addition, the overall maximum temperature, at which the QSHE in the device with *L*_L_ = 1 μm is observed, is not related to the deviation from the expected resistance caused by the increased backscattering in the helical edge channels. Instead, the plateau shifts to lower gate voltages until the bandgap region cannot be probed in the up-sweep for the optimized *V*_min_ anymore (gray region), which is important to optimally apply the gate training. This shift of the whole curve can be attributed to pronounced hysteresis effects arising between the up- and down-sweep during the gate-training process, which limits the applicability of the gate training at elevated temperatures (see fig. S15). The hysteresis originates from charge accumulation at the interface between the gate dielectric and the cap layer of QW heterostructures (*40*, *41*). These hysteresis effects are also the reason why higher temperatures for the 3-μm device are not shown compared to the other devices as the gap region in the up-sweep could not be probed at *T* = 50 and 60 K anymore. We anticipate that with improvements in device quality, especially regarding the stability of the gate, it will be possible to overcome these hysteresis issues. Exploring other capping layers or high-*k* dielectrics such as Al_2_O_3_ or HfO_2_ might also mitigate this issue. In addition to improving the device quality, it is necessary to fabricate QW heterostructures with higher inverted bandgap values for the reliable observation of the QSHE at even higher temperatures. In particular, the bandgap of the InAs/Ga_0.68_In_0.32_Sb/InAs TQW used in this work has a moderate value of 27 meV. By growing InAs/Ga_0.68_In_0.32_Sb/InAs TQWs with slightly different layer thicknesses, the bandgap can be increased up to ~42 meV (see Fig. 1). Moreover, by using the Ga_0.60_In_0.40_Sb alloy and adjusting the buffer materials, which are feasible parameters for high-quality pseudomorphic growth (*51*), the bandgap in the TQW exceeds 50 meV at realistic strain conditions in the layers (see fig. S14). Thus, our experimental results obtained in InAs/GaInSb/InAs TQWs with a moderate bandgap make these QW structures extremely attractive for advancing topological electronics above liquid nitrogen temperatures.

## DISCUSSION

In conclusion, we demonstrated the QSHE up to *T* = 60 K in a material system suitable for device applications. The length-independent quantized resistance values for the gap observed in both local and nonlocal measurement geometries are notable evidence of the QSHE. Using the gate-training technique in devices with a deviating resistance from the expected value, a quantized resistance value could be approached. Furthermore, our temperature study revealed that electronic transport is clearly dominated by helical edge channels with stable quantized resistance up to *T* = 60 K, while observations at even higher temperatures have been prohibited due to pronounced hysteresis effects and the moderate bandgap energy. Considering the continuous improvements in material and device quality and by increasing the bandgap energy, we conclude that achieving the QSHE in this material system at temperatures above that of liquid nitrogen is becoming realistic. Our findings pave the way for developing topological electronics technology at noncryogenic temperatures using III-V semiconductors with well-established growth and processing technologies and enabling integration with silicon technology.

## MATERIALS AND METHODS

### Sample growth and fabrication

The sample was grown by molecular beam epitaxy on an n-doped (001) GaSb substrate, followed by an undoped 200-nm GaSb buffer. Subsequently, a 1500-nm AlSb quasi-substrate was grown to change the lattice constant from GaSb to AlSb. This is followed by a 10× (2.5/2.5) nm GaSb/AlSb superlattice to reduce the dislocation density. The TQW consists of two 9-nm InAs layers separated by a 3.1-nm Ga_0.68_In_0.32_Sb layer and sandwiched between two 40-nm AlSb barriers. A 5-nm GaSb cap was grown on top of the sample to protect it against oxidation. All microscopic Hall bar devices in this study have been fabricated from the same QW heterostructure. For all lithography steps, e-beam lithography was used. As for the etching of the Hall bars, conventional dry-etching techniques were used via reactive ion etching with Ar and Cl. For the ohmic contacts, all antimonide-containing layers are selectively etched using the wet-chemical etchant tetramethylammonium hydroxide (TMAH). The InAs layer is then contacted with ohmic contacts consisting of Cr and Au. As for the gate dielectric, a superlattice of SiO_2_/SiN (5× 10 nm/10 nm and ending with an additional 10-nm SiO_2_ layer) is applied using plasma-enhanced chemical vapor deposition. More information can be found in fig. S1.

### Band structure calculations

Band structure calculations have been performed by using the eight-band k·p Hamiltonian (*23*), which directly considers the interactions between Γ_6_, Γ_8_, and Γ_7_ bands in bulk materials. In the Hamiltonian, we also consider the terms describing the strain effects arising due to mismatch of lattice constants in the buffer, QW layers and AlSb barriers. The calculations have been performed by expanding the eight-component envelope wave functions in the basis set of plane waves and by numerical solution of the eigenvalue problem. More details of calculations and the form of the Hamiltonian can be found in (*23*) and the Supplementary Materials. Parameters for the bulk materials and valence band offsets used in the calculations are taken from (*52*).

## Supplementary Materials

### This PDF file includes:

Supplementary Text Figs. S1 to S15 References
