# Supplementary material for: Quantum spin Hall effect in III-V semiconductors at elevated temperatures: Advancing topological electronics
Source: Sci Adv. 2025 Oct 24;11(43):eadz2408. doi: 10.1126/sciadv.adz2408 (PMC12551694; doi:10.1126/sciadv.adz2408)
Supplement: Supplementary file 1 — Supplementary Text Figs. S1 to S15 References [file sciadv.adz2408_sm.pdf]

Science Advances
AAAS

Supplementary Materials for

Quantum spin Hall effect in III-V semiconductors at elevated temperatures: Advancing topological electronics

Manuel Meyer *et al.*

Corresponding author: Manuel Meyer, manuel.meyer@uni-wuerzburg.de;
Fabian Hartmann, fabian.hartmann@uni-wuerzburg.de

Sci. Adv. **11**, eadz2408 (2025)
DOI: 10.1126/sciadv.adz2408

This PDF file includes:

Supplementary Text
Figs. S1 to S15
References

Layer structure and sample fabrication

The layer structure of the sample investigated is provided in Fig. S1A. The sample was grown by molecular beam epitaxy (MBE) on an n-doped (001) GaSb substrate, followed by an undoped 200 nm GaSb buffer.

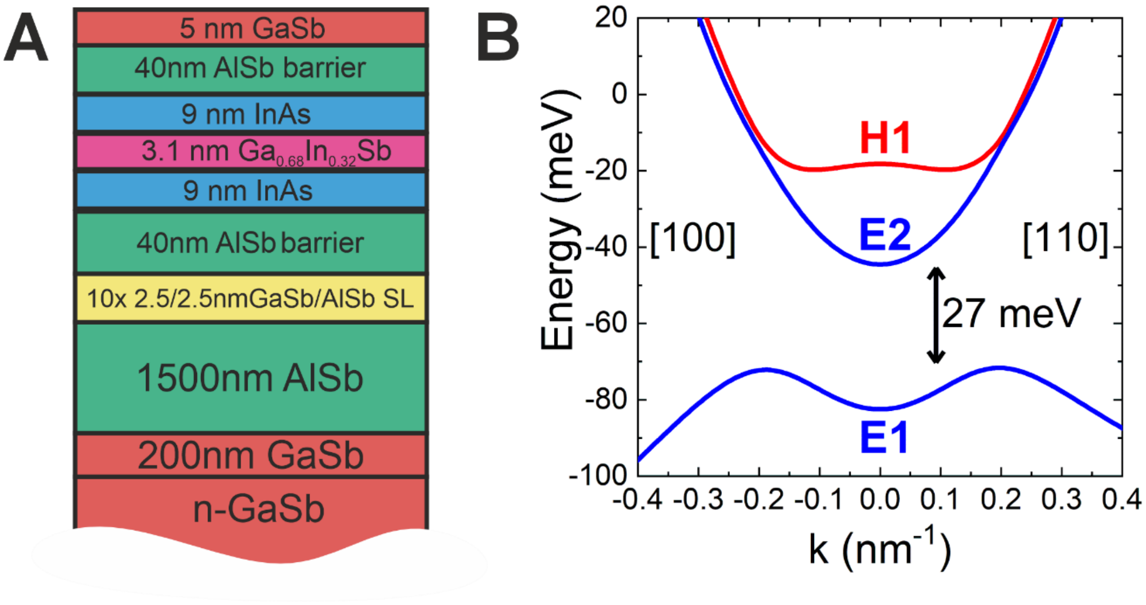

Fig. S1. Layer structure and dispersion of the sample. (**A**) Layer structure of the symmetric InAs/GaInSb/InAs trilayer quantum well (TQW) grown on (001) AlSb buffer. (**B**) Band structure of the grown TQW. The blue and red curves represent the energy-dispersion of the electron-like (E1, E2) and hole-like (H1) subbands, respectively. The positive and negative values of quasimomentum *k* correspond to the [100] and [110] crystallographic orientations. The indirect band gap in TQW is about 27 meV.

Subsequently, a 1500 nm AlSb quasi-substrate was grown to change the lattice constant from GaSb to AlSb. This is followed by a 10x (2.5/2.5) nm GaSb/AlSb superlattice to reduce the dislocation density. The TQW consists of two 9 nm InAs layers separated by a 3.1 nm $Ga_{0.68}In_{0.32}Sb$ layer and sandwiched between two 40 nm AlSb barriers. A 5 nm GaSb cap was grown on top of the sample to protect it against oxidation. All microscopic Hall bar devices in this study have been fabricated from the same QW heterostructure. For all lithography steps, e-beam lithography was used. As for the etching of the Hall bars, conventional dry-etching techniques were used via reactive ion etching with Ar and Cl. For the ohmic contacts, all antimonide-containing layers are selectively etched using the wet-chemical etchant tetramethylammonium hydroxide (TMAH). The InAs layer is then contacted with Ohmic contacts

consisting of Cr and Au. As for the gate dielectric, a superlattice of SiO_2/SiN (5x 10 nm/10nm and ending with an additional 10nm SiO_2 layer) is applied using plasma-enhanced chemical vapor deposition.

Fig. S1B represents the energy band dispersion of the grown symmetrical TQW with an indirect bandgap between the E2 and E1 bands of E_{gap} = 27 meV.

Experimentally extracted band gap value

To confirm the theoretically predicted bandgap value, macroscopic Hall bars were fabricated, and temperature-dependent measurements were performed. The contact separation length of the Hall bar is L_L = 10 µm and the complete length and width are L = 130 µm and W = 20 µm. In Fig. S2A, the longitudinal resistance $R_{03,12}$ versus T = 1.7 K to 130 K is presented. From the Arrhenius plot in Fig. S2B, the band gap energy can be extracted to E_{gap} = (24.2 ± 2.8) meV by applying a linear fit to the high-temperature regime (*19*). This experimental gap value is in good agreement with the one extracted from the calculations of 27 meV.

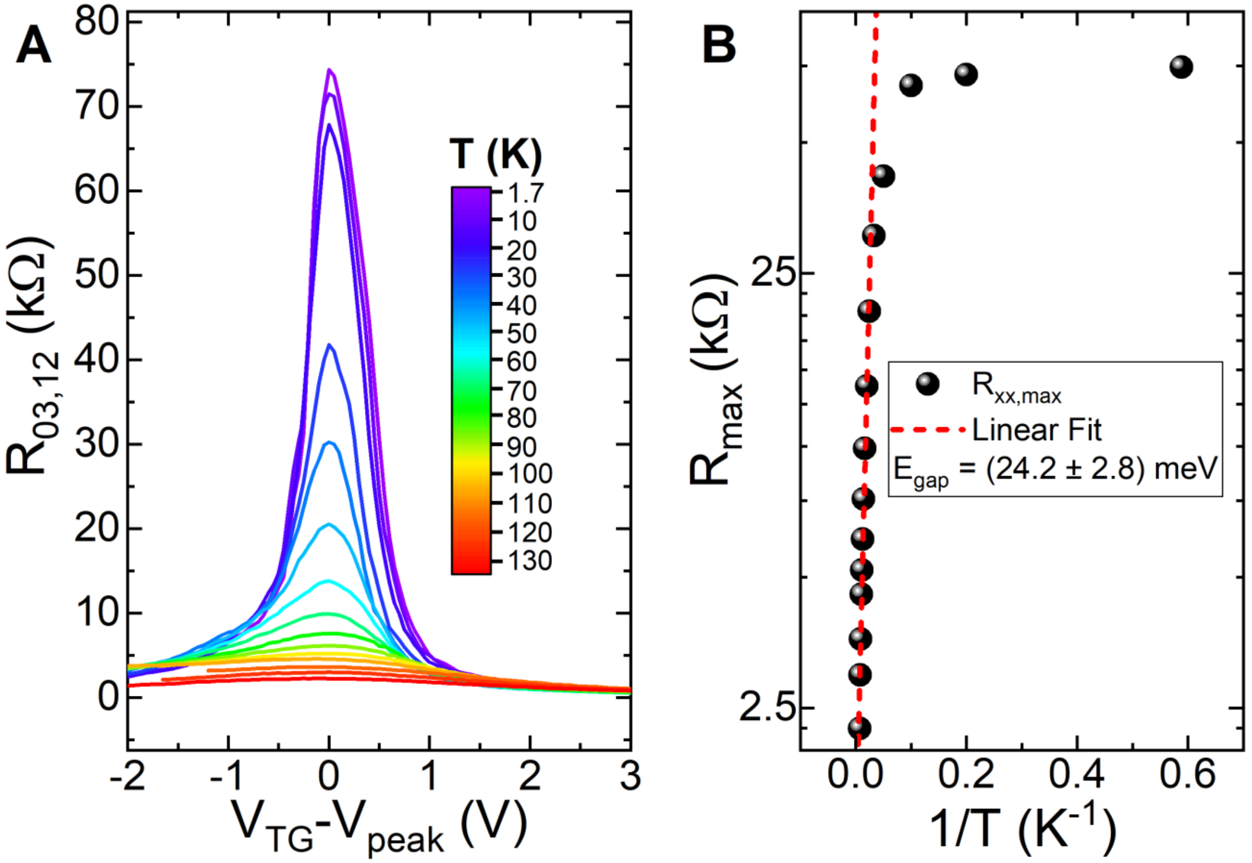

Fig. S2. Temperature-dependent measurements. (**A**) Temperature dependence of the resistance $R_{03,12}$ for T = 1.7 to 130 K for a macroscopic Hall bar. (**B**) Arrhenius plot of the resistance maxima. By applying a linear fit to the high-temperature regime, the band gap can be extracted to E_{gap} = (24.2 ± 2.8) meV.

Expected quantized values in local and nonlocal configurations

In the following, we show that the four-probe resistances $R_{03,12}$ and $R_{01,32}$ are given by $R_{03,12} = h/2e^2$ and $R_{01,32} = h/6e^2$ for helical edge states without backscattering, using the Landauer-Büttiker formalism.

We use the well-known formula for multiterminal conductors (*53*):

$I_p = \sum_q G_{pq}(V_p - V_q)$,

Where I_p is the current flowing from the external circuit into the lead p, V_q is the voltage at the lead q and G_{pq} is the conductance from lead p to lead q. In the simplest case of perfect ballistic helical edge states,

for the geometry indicated in Figure S8, the conductances G_{pq} are given by the elements of the conductance matrix:

$$G = \begin{bmatrix} 0 & 1 & 0 & 0 & 0 & 1 \\ 1 & 0 & 1 & 0 & 0 & 0 \\ 0 & 1 & 0 & 1 & 0 & 0 \\ 0 & 0 & 1 & 0 & 1 & 0 \\ 0 & 0 & 0 & 1 & 0 & 1 \\ 1 & 0 & 0 & 0 & 1 & 0 \end{bmatrix}, \qquad (1)$$

where the unit of conductance has been taken equal to 1. Let us calculate the resistances for a current I_{03} flowing from lead 0 to lead 3. Setting $V_3 = 0$ and leaving out the row and column corresponding to lead 4, we get the linear system:

$$\begin{bmatrix} I_{03} \\ 0 \\ 0 \\ 0 \\ 0 \end{bmatrix} = \begin{bmatrix} 2 & -1 & 0 & 0 & -1 \\ -1 & 2 & -1 & 0 & 0 \\ 0 & -1 & 2 & -1 & 0 \\ 0 & 0 & -1 & 2 & -1 \\ -1 & 0 & 0 & -1 & 2 \end{bmatrix} \begin{bmatrix} V_0 \\ V_1 \\ V_2 \\ V_4 \\ V_5 \end{bmatrix}, \qquad (2)$$

From which all the voltages of the different probes are easily calculated. From equation (2), the resistance $R_{03,12}$ can be calculated to $R_{03,12} = (V_2 - V_1) / I_{03} = 1/2$ (Fig. S3A). A similar calculation can be done for $R_{01,32}$. It yields $R_{01,32} = (V_3 - V_2) / I_{01} = 1/6$ (Fig. S3B).

Such calculations can be performed for much more complex devices, introducing 'virtual contacts' between the real ones, or assuming some backscattering between the 'real' leads.

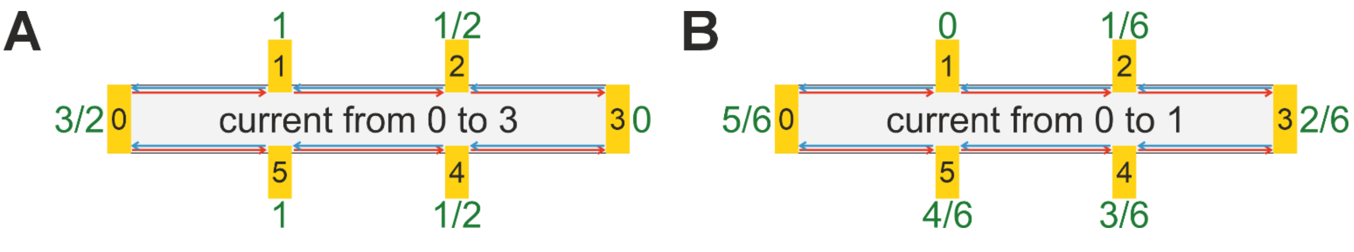

Fig. S3. Expected quantized resistance values. Scheme of a Hall bar with contacts labeled from 0 to 5 and the helical edge states are shown as red and blue arrows. The voltages are indicated in green, in units of $\boldsymbol{h/e^2}$. (**A**) The current flows from lead 0 to lead 3. (**B**) The current flows from lead 0 to lead 1.

Reproducibility of the peak positions

To confirm the reproducibility of the peak position, experiments in local configuration $R_{03,12}$ with L_L = 3 µm and nonlocal configuration $R_{01,32}$ with L_{NL} = 3.5 µm have been performed. These are depicted in Figs. S4A and B, respectively. Three measurements for each configuration were conducted for the same gate-voltage range (V_{TG} = +3 to -3 V). The resistance peaks always remain at the same position of $V_{TG} \approx$ -1.47 V.

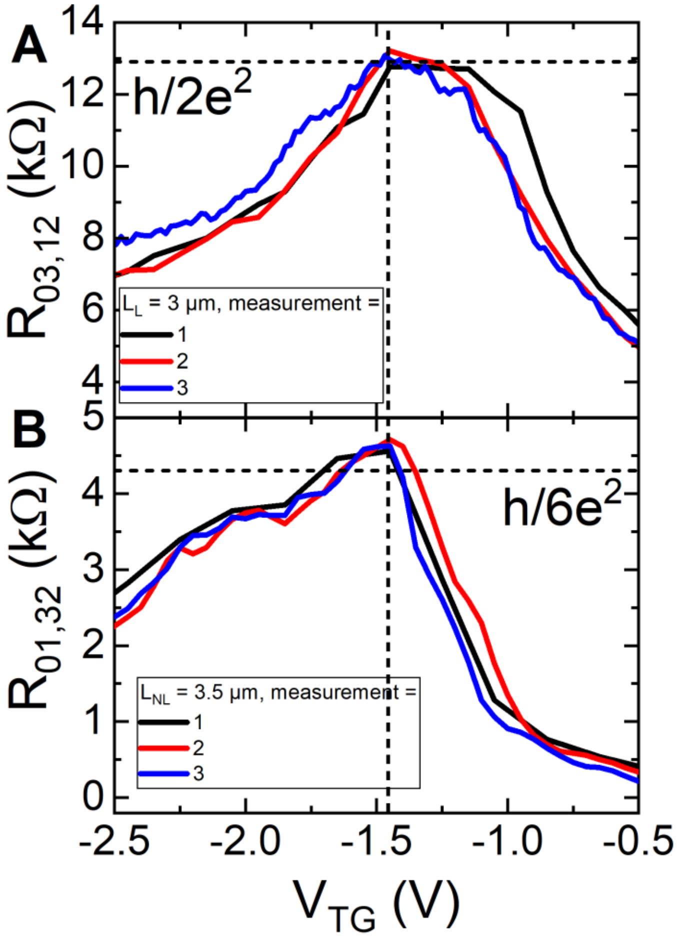

Fig. S4. Peak position in local and nonlocal configurations. (**A**) Peak position of the same device for the local configuration $R_{03,12}$ with L_L = 3 µm and (**B**) for the nonlocal configuration $R_{01,32}$ with L_{NL} = 3.5 µm. For both configurations, the measurements have been performed three times, and the peak can always be found at $V_{TG} \approx$ -1.47 V.

Additional measurement configurations

As shown before, dependent on the measurement configuration (i.e., where the current is applied and the voltage is measured), different quantized resistance values are expected. In Fig. S5A, the current is applied between contacts 1 and 5 and the resistance is measured between 2 and 3. The resistance value for the gap is in good agreement with the expected value of $h/3e^2$. In Fig. S5B, the current is applied between contacts 0 and 5 and the voltage is either measured between 2 and 3 or 3 and 4. In both cases, the expected quantized value of $h/6e^2$ again coincides with the maximum resistance in the gap region. These additional configurations are further evidence for the QSHE.

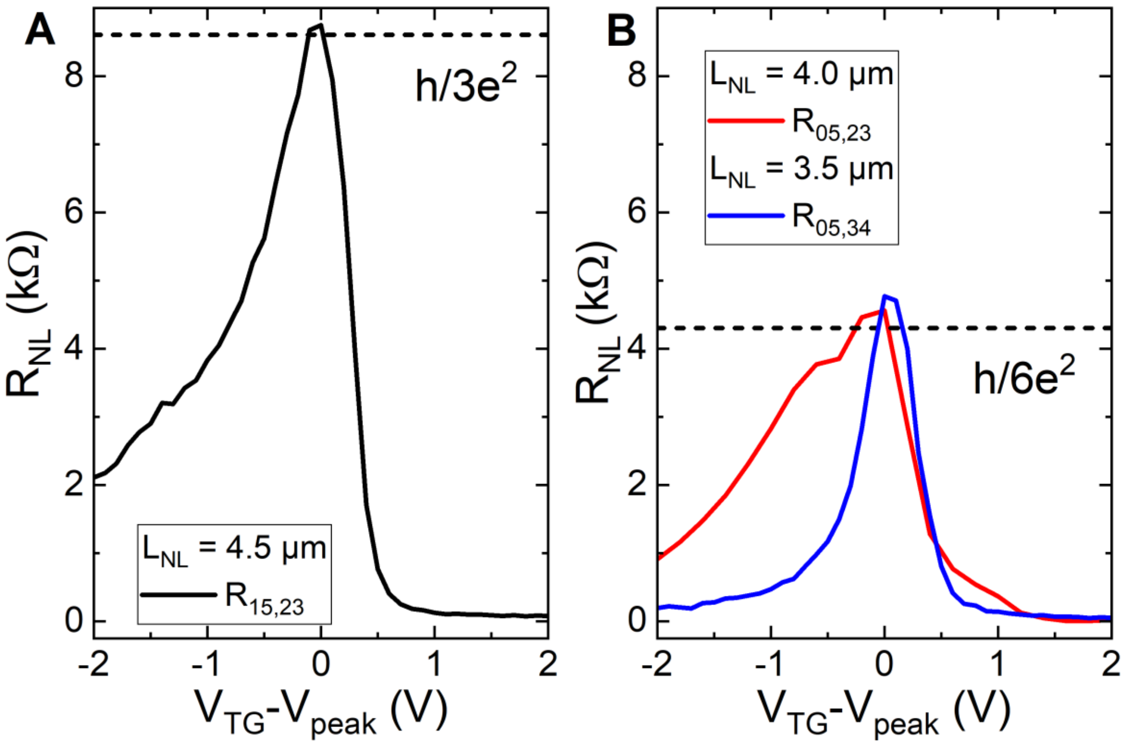

Fig. S5. Additional quantized resistance values in different configurations. (**A**) $R_{15,23}$ as a function of V_{TG} for a device with L_{NL} = 4.5 µm. The maximum resistance in the gap is in good agreement with the expected value of $h/3e^2$. (**B**) $R_{05,23}$ and $R_{05,34}$ as a function of V_{TG} for a device with L_{NL} = 4.0 µm and 3.5 µm, respectively. The maximum resistances are in both cases in good agreement with the expected value of $h/6e^2$.

Separating bulk and edge contributions

To observe the QSHE, trivial parasitic contributions need to be negligible. In InAs/GaSb BQWs, the low band gap (*30, 18*) and native p-doping (*54*) of antimonides resulted in a large residual bulk contribution and impeded the observation of the QSHE. The bulk resistance of BQWs or TQWs could be improved by doping the interface between InAs and GaSb with Si (*19*) or by growing GaInSb instead of GaSb (*38, 36, 39*). The question remains how much this improves the bulk insulation and if the current flows solely at the edges due to the QSHE. Therefore, we performed a multi-probe analysis on macroscopic devices to separate bulk and edge contributions. Detailed information about this method can be found in Ref. (*46*). Briefly, this technique consists in the measurement of 45 four-probe resistances as a function of top-gate voltage. Then, for each gate voltage of interest, the 45 resistances are fitted with only two parameters, the bulk conductivity (in µS/sq, sq = 1 µm × 1 µm) and the edge conductance (in µS/µm). The edge conductivity G_e and bulk conductivity σ_B for two different devices HB10a and HB70a are shown in Fig. S6A and B, respectively. The dimensionalities of the Hall bars are W = 20 µm and L_L = 10 and 70 µm for HB10a and HB70a, respectively. From the analysis of the HBs in the band gap, one observes that the bulk conductivity becomes negligibly small, indicating an insulating bulk with a resistivity of approx. 2 MΩ/sq for HB10a and even more than 100 MΩ/sq for HB70a.

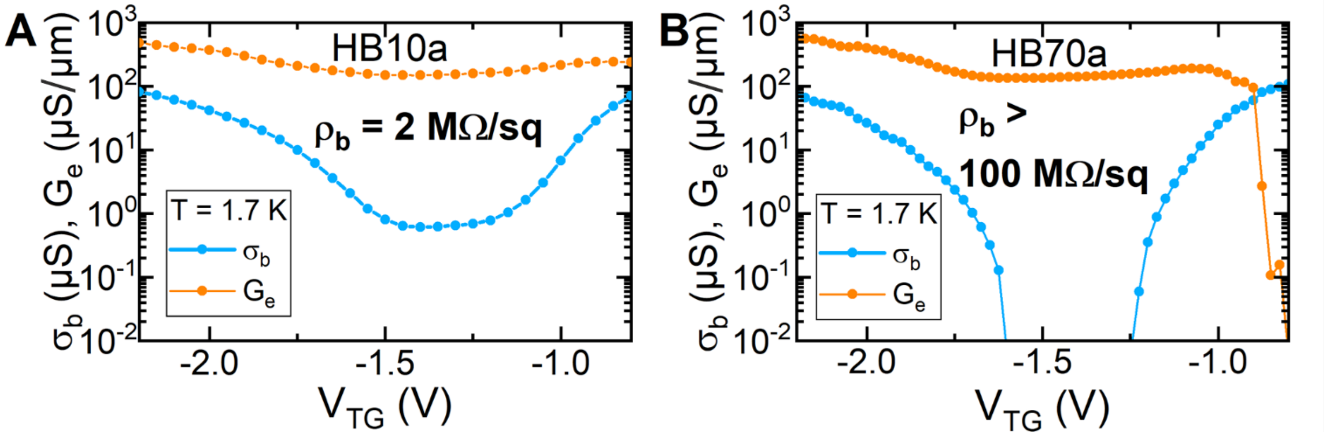

Fig. S6. Bulk and edge contributions of macroscopic Hall bars. Evolution of the edge conductance G_e (orange dots) and bulk conductance σ_b (blue dots) as a function of top-gate voltage V_{TG} for HB10a (L_L = 10μm) in (**A**) and HB70a (L_L = 70 μm) in (**B**), respectively. In the gap, (around V_{TG} = -1.2V) the bulk conductance is diminished, and the transport is dominated by the edges.

The difference of these bulk resistivity values can be explained by a variation of the number of defects. In general, the residual bulk conductivity in non-intentionally doped GaSb-based materials grown by MBE results mostly from native Ga antisite defects and/or Ga vacancy p-doping defects (*54–56*), whereas the AlSb quasi substrate is insulating (*57*). Additional conductivity paths may arise from dislocations due to the growth on the AlSb buffer, as dislocations are known to generate carrier leakage in III-V semiconductor heterostructures (*58–60*). For both devices, the bulk conductance is diminished, and the transport is dominated by the edges. With G_e = 150 μS/μm for both devices, one can extract the phase coherence length to $\lambda = (G_e h/e^2) \approx 3.9$ μm (*46*), which is in good agreement with the value extracted from Fig. 2C from the manuscript.

Determination of the phase coherence length

To accurately extract the phase coherence length λ, we performed a multi-probe analysis (*46*) on a few Hall bar devices to extract the edge resistance R_{edge} as a function of contact separation length. In Fig. S7A we show the (local) longitudinal resistances $R_{03,12}$ measured in HB10a at T = 5 K for the down sweep (in

blue) and up sweep (in green). The resistance depends on the direction of the gate voltage sweep. From Fig. S6, we know that the bulk conductivity is negligible at these low temperatures. Hence, the maxima of the $R_{03,12}$ peaks already give a first estimate of the backscattering length. Using $R_{03,12} \simeq \frac{h}{2e^2} L/\lambda$, a first estimation yield $\lambda \simeq 3 - 5$ µm. For more precise determination of λ, we performed four-probe resistance measurements in all 45 geometries as a function of top gate voltage. Then the 45 maxima of the resistance peaks were fitted with six resistances R_{edge} of the six edges, as independent parameters. The edge lengths are 10 µm (two edges) and 50 µm (four edges) for HB10a. The fits are very good, even if one considers the value of the reduced chi-square – it means that the model is meaningful. The six obtained edge resistances are reported in Fig. S7B for the two directions of the gate voltage sweeps. From these resistances, the backscattering length can be fitted as $R_{edge} = \frac{h}{e^2}\frac{L}{\lambda}$, which gives $\lambda \simeq 4 - 6$ µm. All these values suggest that a quantization should be observed in micrometer-sized devices. Note that since the gate training method was not applied during this experiment, one can expect a larger λ after gate training as it is already indicated by the difference between the two sweep directions.

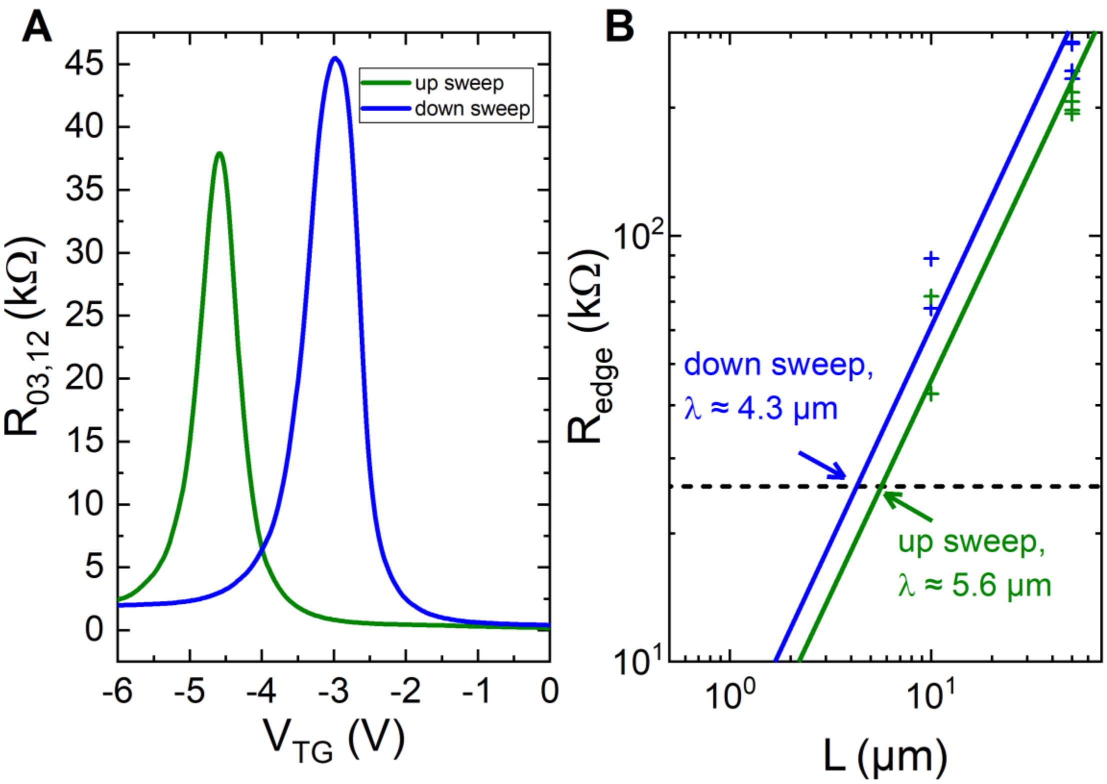

Fig. S7. Edge resistances for different lengths for the macroscopic device HB10a measured at *T* = 5 K. (**A**) Longitudinal resistance $R_{03,12}$ for the down sweep (in blue) and up sweep (in green), respectively. (**B**) The symbols are the results of the 6-parameter fits for the edge resistance in dependence of the edge lengths.

The same analysis was applied to other devices (4 in total). We therefore obtain the edge resistances for different lengths of the different devices, as shown in Fig. S8. The green triangles are the extracted edge resistances from the data, and the solid line is the best fit with $R_{edge} = \frac{h}{e^2}\frac{L}{\lambda}$. We emphasize that λ in Figs. S7B and S8 is obtained at the intersection of the fit with h/e^2 and not $h/2e^2$ as the fit gives the edge resistances separately. We get $\lambda \approx 4.7$ μm, a value greater than all the edge lengths of the microscopic devices.

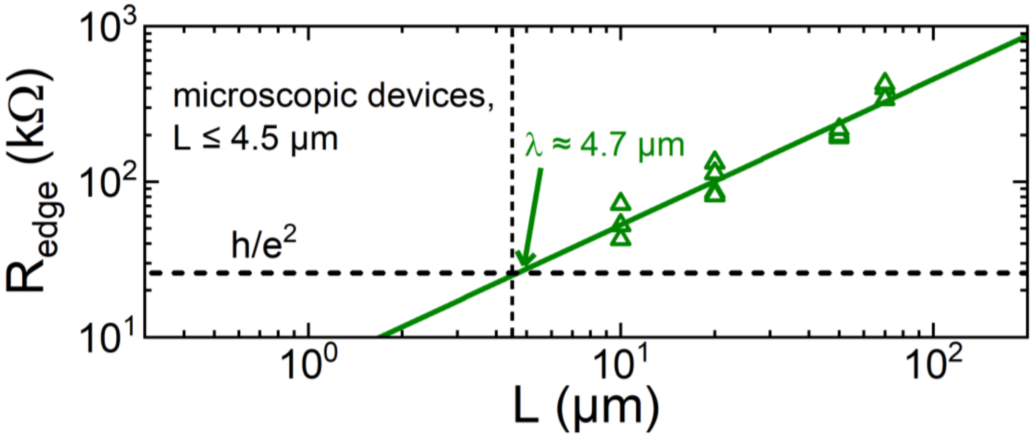

Fig. S8. Edge resistances versus length for macroscopic devices. R_{edge} extracted from a six-parameter fit as a function of edge length for several Hall bar devices. The intersection with h/e^2 gives the phase coherence length $\lambda \approx 4.7$ µm.

Remarkably, the gate training technique was also not even used in these experiments. One might therefore expect an even larger λ after gate training.

Breaking of time-reversal symmetry and helical edge states

The robustness of the QSHE in the presence of magnetic field strongly depends on whether a 2D system possesses spatial inversion symmetry, as well as on the orientation of the applied magnetic field. Indeed, the presence of an inversion center leads to the conservation of physical spin S_z, where z is the axis perpendicular to the plane of the 2D system. In this case, the polarization of the edge states is also oriented along the z axis; and the application of a perpendicular magnetic field does not lead to the breaking of the QSHE until the field exceeds a critical value B_c, in which the zero-mode Landau level cross (*61*). On the contrary, the application of even small magnetic fields in the 2D system plane leads to the gap opening at the Dirac point of the edge states. If the Dirac point of the edge states is located inside the band gap of the bulk states, then this leads to the breaking of the QSHE. However, if the Dirac point is buried inside conduction or valence band of the bulk states, the quantum edge transport remains robust even in the presence of in-plane magnetic field (*62*). Importantly, the presence of inversion asymmetry yields the gap opening for the edge states already in a perpendicular magnetic field. While an applied vertical electric

field through the top gate breaks structure inversion asymmetry (SIA) also in the TQWs, in the gap region the electric field is rather small. Figure S9 provides measurements of the longitudinal resistance $R_{03,12}$ under small magnetic field applied in-plane (Fig. S9A) and out-of-plane (Fig. S9B) of the TQW at low temperatures.

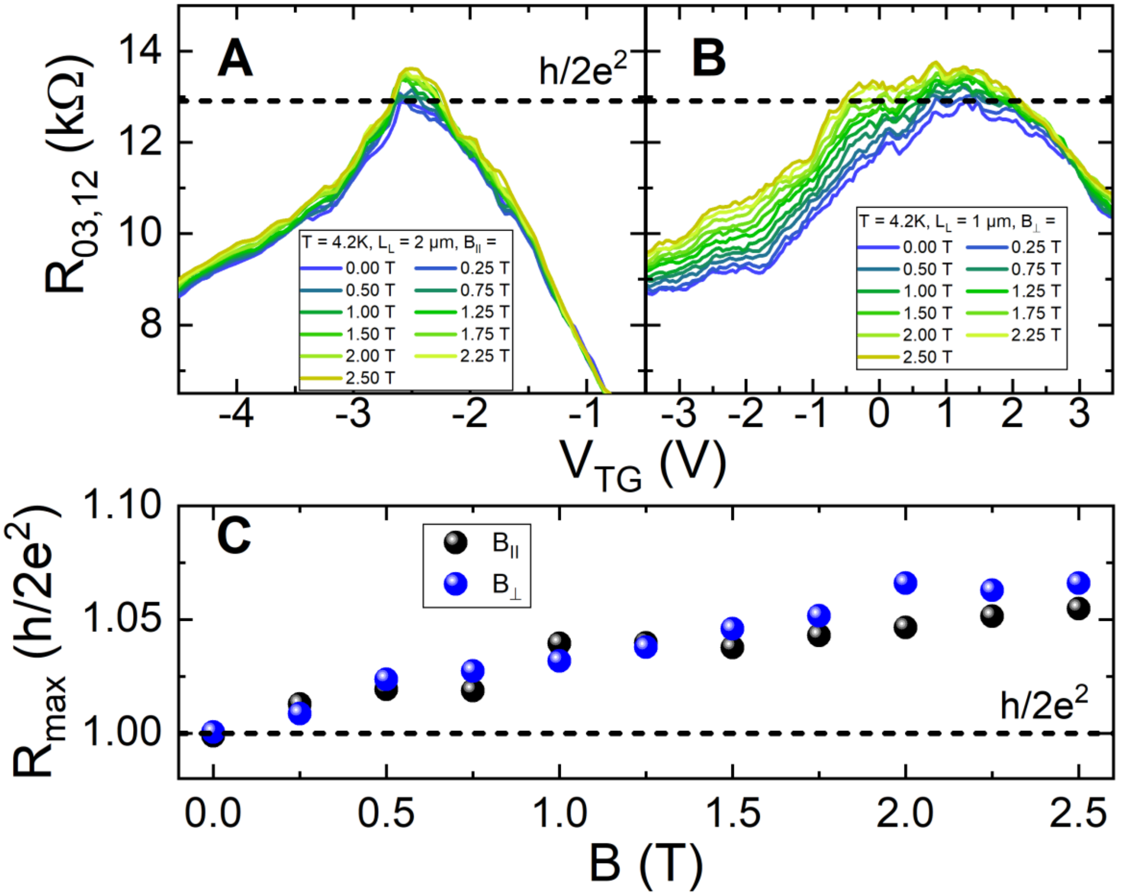

Fig. S9. Time-reversal symmetry breaking in microscopic devices. Longitudinal resistance $R_{03,12}$ as a function of top-gate voltage V_{TG} at $T = 4.2$ K for (**A**) in-plane magnetic field $B_{||}$ (parallel to the current) and (**B**) perpendicular magnetic field $B_\perp$. (**C**) The resistance maxima as a function of magnetic field for both field configurations.

As seen in Fig. S9C, the maximum resistance in the band-gap region increases with magnetic field that indicates the gap opening for the edge states, suppressing of the quantized values of the QSHE for both

magnetic field configurations. The latter evidences a pronounced role of the bulk inversion asymmetry (BIA) and the interface inversion asymmetry (IIA) in the symmetric InAs/GaInSb/InAs TQW. We note that a similar behavior of the longitudinal resistance, but with a more pronounced effect of change at the same values of the magnetic field was also observed in an InAs/GaInSb BQWs (*36*). The stronger change in $R_{03,12}$ may indicate the larger g-factor values for the edge electrons due to the presence of SIA inherent in InAs/GaInSb BQWs. Alternatively, considering the band structure of the grown sample, the weakness of the magnetic field effect on $R_{03,12}$ may be because the Dirac point of the edge states is hidden inside either the conduction E2 subband or the valence E1 subband (see Fig. S10A). In this case, the effect of the magnetic field on longitudinal resistance is expected to be substantially stronger for the sample with the maximum band gap, for which the E2 and H1 subbands are located close in energy (see Fig. S10B).

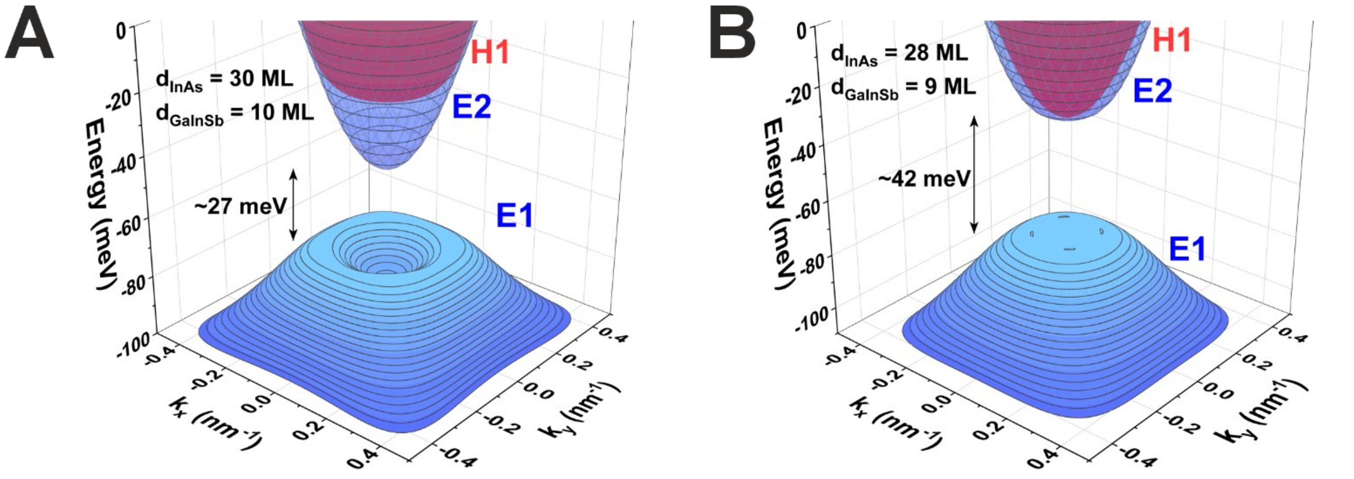

Fig. S10. 3D dispersion of the investigated sample and of the highest band gap. 3D plots of the band structure for the sample from the main text in (**A**) and the sample with the highest possible band gap for $Ga_{0.68}In_{0.32}Sb$ on AlSb in (**B**). The *x* and *y* axes are oriented along [100] and [010] crystallographic directions, respectively.

Indeed, calculations (see Fig. S11) show that the Dirac point of the helical edge states is likely buried within the conduction E2 subband rather than lying within the bulk gap. In real devices, although boundary

conditions may vary, it is reasonable to assume that the Dirac point remains hidden inside the bulk band, which reduces the magnetic field's ability to open a sizable gap at the Dirac point and thus suppressing edge conduction.

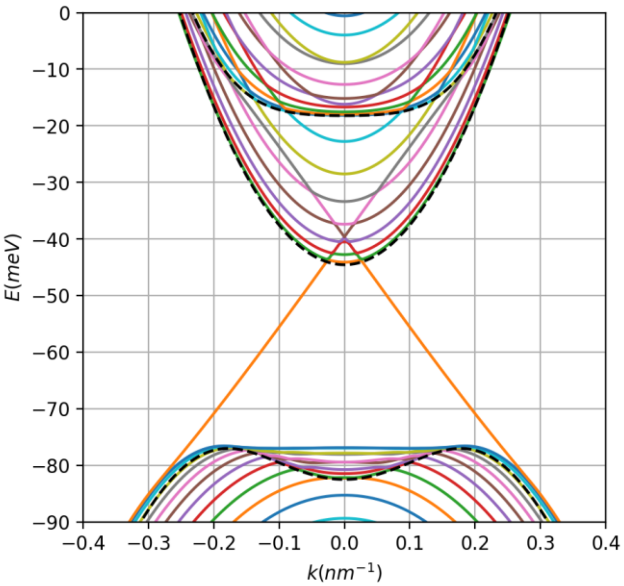

Fig. S11. Buried Dirac point. Band dispersion for a nanoribbon made from the same trilayer structure (solid lines: nanoribbon; dashed black lines: bulk dispersion). The Dirac point is buried in the conduction band. Calculation performed with the kwant package (tight binding calculations).

To clarify the reasons for such a weak effect of the magnetic field on $R_{03,12}$ in our devices compared to the previously studied BQWs is beyond the scope of this paper and will be the subject of future investigations. Nevertheless, the small suppression of quantized $R_{03,12}$ values by the magnetic field in microscopic Hall bar devices shown in Fig. S9 can be still considered as a breaking of TRS by magnetic field.

Model of the edge resistance with additional backscattering

We assume now that the edge resistance between two leads separated by a length L is given by the simple formula:

$$R_e(L) = \max\left(1, \frac{L}{\lambda}\right), \tag{3}$$

where λ is the phase coherence length. This formula mimics the saturation of the resistance in the shortest edges, as well as their ohmic behavior in the longest edges. The conductance matrix is then given by:

$$G= \begin{bmatrix} 0 & R_e(L_{NL}) & 0 & 0 & 0 & R_e(L_{NL}) \\ R_e(L_{NL}) & 0 & R_e(L_L) & 0 & 0 & 0 \\ 0 & R_e(L_L) & 0 & R_e(L_{NL}) & 0 & 0 \\ 0 & 0 & R_e(L_{NL}) & 0 & R_e(L_{NL}) & 0 \\ 0 & 0 & 0 & R_e(L_{NL}) & 0 & R_e(L_L) \\ R_e(L_{NL}) & 0 & 0 & 0 & R_e(L_L) & 0 \end{bmatrix}, \tag{4}$$

The results of the calculation of the local and non-local resistances for different geometries are given in Figure S12. At $\frac{1}{\lambda} \simeq 0$, all resistances are quantized. Increasing $\frac{1}{\lambda}$, the nonlocal resistance $R_{01,32}$ loses its quantization 1/6 first for devices L_L=1 (the largest L_{NL}), then for device L_L=2 and finally for device L_L= 3 µm. The resistance quantization is more robust in the local configuration $R_{03,12}$, but is also lost when $L_L$exceeds λ. It is the most robust for L_L = 1 µm.

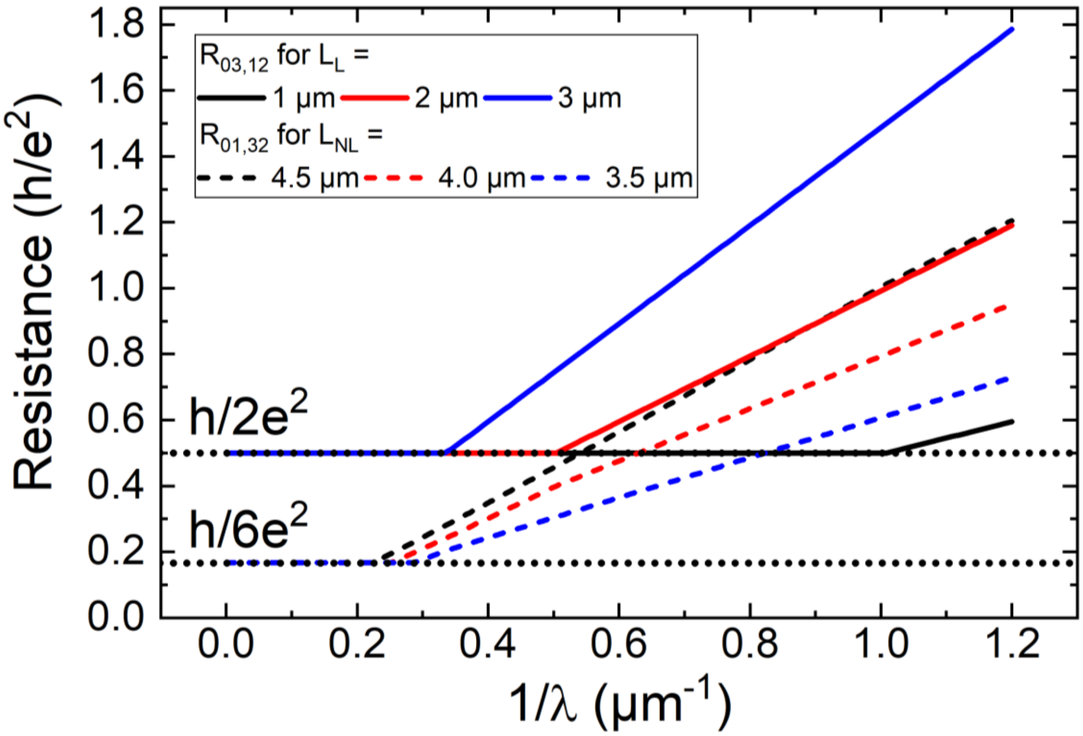

Fig. S12. Edge resistances in local and nonlocal configurations with additional backscattering. Resistance as a function of $1/\lambda$ in local and nonlocal configurations. For the nonlocal configuration ($R_{01,32}$), the quantized value of the resistance is first lost for L_L = 1µm (L_{NL} = 4.5 µm), then for L_L = 2 µm (L_{NL} = 4.0 µm) and at last for L_L = 3 µm (L_{NL} = 3.5 µm). In the local configuration ($R_{03,12}$), the quantized value of the resistance is more robust and is at last lost for L_L = 1 µm.

Mobility and mean free path

One can also expect a quantized resistance for 1D edge channels if the contact separation length L between the voltage probes is smaller than the mean free path λ_{mfp}. This would require 1D edge channels in our 2D system even without the quantum spin Hall effect (QSHE), which is unlikely. However, we still want to exclude this possibility of the quantization for $L < \lambda$ for our devices studied in the main text. Figure S13 shows an additional Hall measurement for the Hall bar device with L_L = 2 µm from Figure 2 in the main text. From this measurement, the charge carrier density n, mobility μ and $\lambda_{mfp} = \frac{\hbar}{e}\mu\sqrt{2\pi n}$ (*20*) was extracted at a given top-gate voltage V_{TG} = +10 V, which yield $n = 9.31 \times 10^{11}$ cm^{-2}, $\mu = 13.4 \times 10^3$ cm^2/Vs and λ_{mfp} = 107 nm. These values are extracted when the Fermi energy lies deep in the conduction band and even there λ_{mfp} is smaller than all the contact separation lengths. In the band gap, where the helical

edge channels appear, the mean free path is expected to be much smaller because of smaller charge carrier densities and mobilities. Therefore, the observed quantized resistances in the main text are attributed to the observation of the QSHE. The overall low mobility also indicates a larger bulk resistivity.

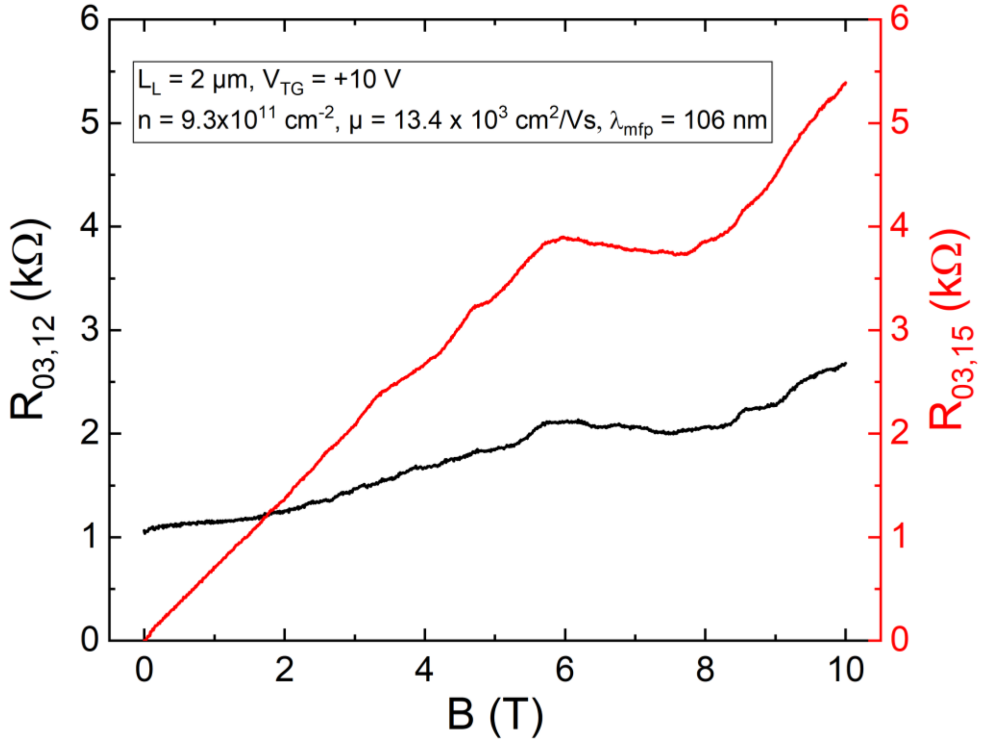

Fig. S13. Extraction of charge carrier density, mobility and mean free path. Longitudinal resistance $R_{03,12}$ and Hall resistance $R_{03,15}$ as a function of the magnetic field at V_{TG} = +10 V for the Hall bar device with L_L = 2 µm from Figure 2 from the main text. λ_{mfp} is smaller than all the contact lengths.

Band structure calculations and maximum inverted band gap of symmetric InAs/GaInSb/InAs TQWs grown on AlSb buffer

Band structure calculations in the main text have been performed by using the eight-band k·p Hamiltonian (*23*), which directly considers the interactions between Γ_6, Γ_8 and Γ_7 bands in bulk materials. In the Hamiltonian, we also consider the terms describing the strain effects arising due to mismatch of lattice constants in the buffer, QW layers and AlSb barriers. The calculations have been performed by expanding the eight-component envelope wave functions in the basis set of plane waves and by numerical solution

of the eigenvalue problem. Details of calculations and the form of the Hamiltonian can be found in Ref. (*23*). Parameters for the bulk materials and valence band offsets used in the calculations are taken from Ref. (*52*).

As mentioned in the main text, higher inverted band gap values can potentially be achieved in InAs/GaInSb/InAs TQWs by increasing the In composition of the GaInSb alloy. To date, high-quality pseudomorphic growth has been achieved for InAs/$Ga_{1-x}In_xSb$-based heterostructures with $x = 0.40$ (*51*). Therefore, we also further consider three-layer InAs/$Ga_{0.60}In_{0.40}Sb$ QWs with the same In content. Even though previous theoretical studies predict the implementation of a band gap for the TI state in InAs/$Ga_{0.60}In_{0.40}Sb$ TQWs of 60 meV grown on specific buffer (*25*), the implementation of a TQW with such a gap value is not possible due to the excessively high strain arising in the QW layers. Therefore, we further restrict ourselves to the more realistic case of InAs/$Ga_{0.60}In_{0.40}Sb$ TQWs grown on (001) AlSb buffer. Recently, InAs/$Ga_{0.60}In_{0.40}Sb$ QWs have been studied in BQW geometry (*38*). Figs. S14A and B summarize our calculations performed for realistic InAs/$Ga_{0.60}In_{0.40}Sb$ TQWs. For realistic values of the layer widths, the band gap in the TI state for this TQW can reach 50 meV, which is nearly 2 times larger than the band gap of the sample studied in the main text.

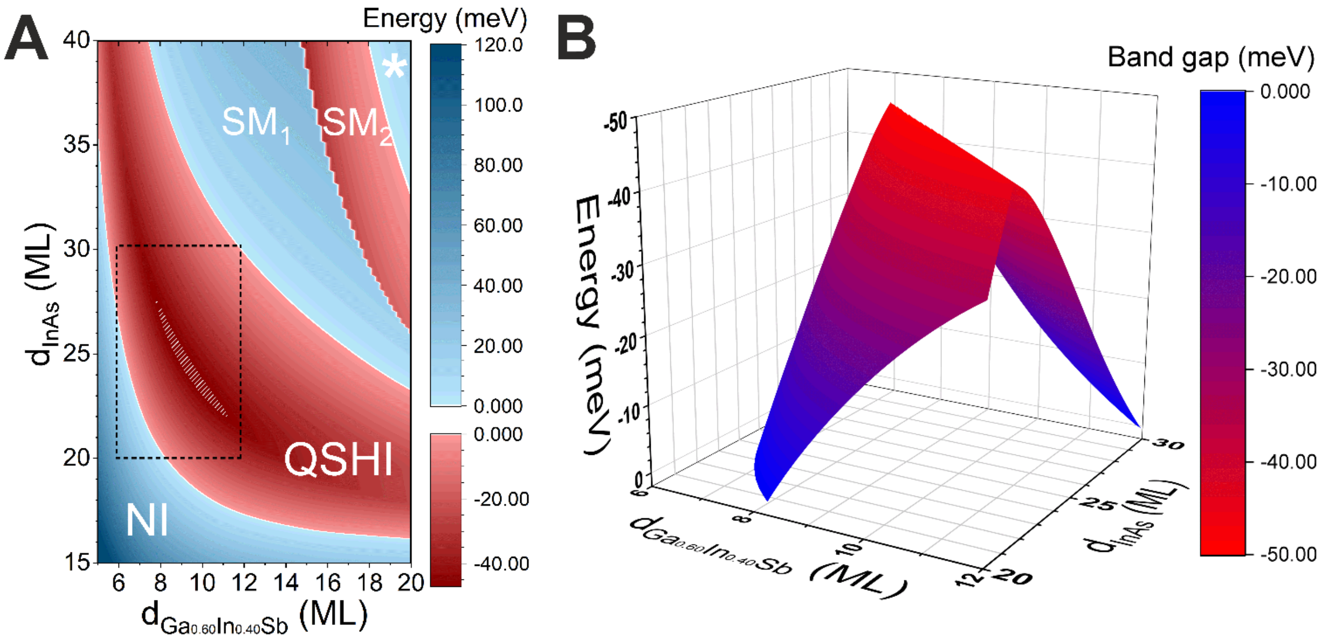

Fig. S14. Maximum band gap for inverted InAs/$Ga_{0.60}In_{0.40}Sb$/InAs TQWs. (**A**) Colormap diagram of symmetric InAs/$Ga_{0.60}In_{0.40}Sb$/InAs TQWs grown on (001) AlSb buffer. NI and QSHI regions are marked, while the white region represents the area where the band gap is close to its maximum value. The regions SM_1 and SM_2 confirm the semimetals with single and double band inversions, respectively. The energy for these SM states represents the overlapping between conduction and valence bands. The blue region marked by asterisk corresponds to higher-order insulator state (*63*). Here, 1 ML corresponds to half of the lattice constant of the bulk material. (**B**) Band gap as a function of the layer widths of InAs/$Ga_{0.60}In_{0.40}Sb$ TQWs in the rectangular region marked by dotted edges in panel (**A**).

Hysteresis width with increasing temperatures

As the temperature increases, the hysteresis width ΔV (difference in the peak positions for both gate voltage sweep directions) also increases. Figure S15 shows ΔV as a function of temperature for the three devices presented in Fig. 4 of the manuscript. With increasing temperature, the up-sweep peak (used in the gate-training procedure) shifts to lower gate voltages, and the hysteresis width grows. At certain temperatures, the gate-trained up-sweep peak moves outside the accessible gate-voltage range, making gate training no longer applicable. These issues originate from the specifics of sample growth and fabrication. They might be mitigated by utilizing different capping layers and/or gate dielectrics.

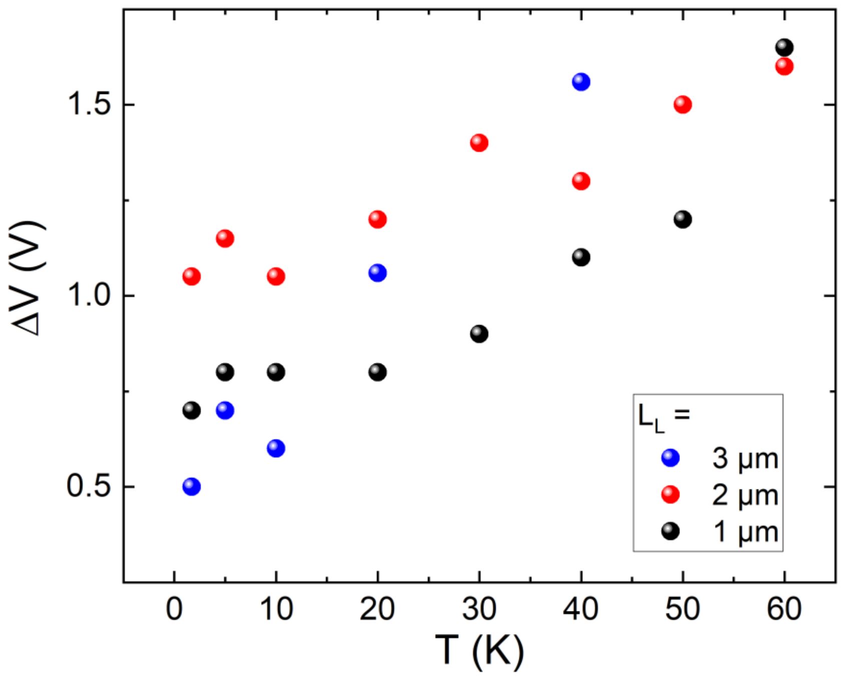

Fig. S15. Temperature dependence of the hysteresis width. Hysteresis width *ΔV* as a function of temperature for the three devices from Fig. 4 of the manuscript. *ΔV* increases with temperature impeding the observation of the QSHE at even higher temperatures.

In addition to hysteresis, the moderate band gap and the resulting thermally activated carriers leading to residual bulk conductivity also limit high-temperature performance.

REFERENCES AND NOTES

1. C. L. Kane, E. J. Mele, Quantum spin Hall effect in graphene. *Phys. Rev. Lett.* **95**, 226801 (2005).

2. C. L. Kane, E. J. Mele, Z_2 topological order and the quantum spin Hall effect. *Phys. Rev. Lett.* **95**, 146802 (2005).

3. L. Sheng, D. N. Sheng, C. S. Ting, F. D. M. Haldane, Nondissipative spin Hall effect via quantized edge transport. *Phys. Rev. Lett.* **95**, 136602 (2005).

4. B. A. Bernevig, S. C. Zhang, Quantum spin Hall effect. *Phys. Rev. Lett.* **96**, 106802 (2006).

5. M. Z. Hasan, C. L. Kane, Colloquium: Topological insulators. *Rev. Mod. Phys.* **82**, 3045–3067 (2010).

6. B. A. Bernevig, T. L. Hughes, S. C. Zhang, Quantum spin Hall effect and topological phase transition in HgTe quantum wells. *Science* **314**, 1757–1761 (2006).

7. M. König, S. Wiedmann, C. Brüne, A. Roth, H. Buhmann, L. W. Molenkamp, X. Qi, S. Zhang, Quantum spin Hall insulator state in HgTe quantum wells. *Science* **318**, 766–770 (2007).

8. A. Roth, C. Brüne, H. Buhmann, L. W. Molenkamp, J. MacIejko, X. L. Qi, S.-C. Zhang, Nonlocal transport in the quantum spin Hall state. *Science* **325**, 294–297 (2009).

9. C. Brüne, A. Roth, H. Buhmann, E. M. Hankiewicz, L. W. Molenkamp, J. MacIejko, X. L. Qi, S. C. Zhang, Spin polarization of the quantum spin Hall edge states. *Nat. Phys.* **8**, 485–490 (2012).

10. M. G. Vergniory, L. Elcoro, C. Felser, N. Regnault, B. A. Bernevig, Z. Wang, A complete catalogue of high-quality topological materials. *Nature* **566**, 480–485 (2019).

11. H. Zhang, C. X. Liu, X. L. Qi, X. Dai, Z. Fang, S. C. Zhang, Topological insulators in Bi_2Se_3, Bi_2Te_3 and Sb_2Te_3 with a single Dirac cone on the surface. *Nat. Phys.* **5**, 438–442 (2009).

12. F. Reis, G. Li, L. Dudy, M. Bauernfeind, S. Glass, W. Hanke, R. Thomale, J. Schäfer, R. Claessen, Bismuthene on a SiC substrate: A candidate for a high-temperature quantum spin Hall material. *Science* **357**, 287–290 (2017).

13. S. Tang, C. Zhang, D. Wong, Z. Pedramrazi, H. Z. Tsai, C. Jia, B. Moritz, M. Claassen, H. Ryu, S. Kahn, J. Jiang, H. Yan, M. Hashimoto, D. Lu, R. G. Moore, C. C. Hwang, C. Hwang, Z. Hussain, Y. Chen, M. M. Ugeda, Z. Liu, X. Xie, T. P. Devereaux, M. F. Crommie, S. K. Mo, Z. X. Shen, Quantum spin Hall state in monolayer 1T'-WTe 2. *Nat. Phys.* **13**, 683–687 (2017).

14. J. J. Zhou, W. Feng, C. C. Liu, S. Guan, Y. Yao, Large-gap quantum spin Hall insulator in single layer bismuth monobromide Bi_4Br_4. *Nano Lett.* **14**, 4767–4771 (2014).

15. C. Liu, T. L. Hughes, X. L. Qi, K. Wang, S. C. Zhang, Quantum spin Hall effect in inverted type-II semiconductors. *Phys. Rev. Lett.* **100**, 236601 (2008).

16. K. Hatsuda, H. Mine, T. Nakamura, J. Li, R. Wu, S. Katsumoto, J. Haruyama, Evidence for a quantum spin Hall phase in graphene decorated with Bi_2Te_3 nanoparticles. *Sci. Adv.* **4**, eaau6915 (2018).

17. S. Wu, V. Fatemi, Q. D. Gibson, K. Watanabe, T. Taniguchi, R. J. Cava, P. Jarillo-Herrero, Observation of the quantum spin Hall effect up to 100 kelvin in a monolayer crystal. *Science* **359**, 76–79 (2018).

18. I. Knez, R.-R. Du, G. Sullivan, Evidence for helical edge modes in inverted InAs/GaSb quantum wells. *Phys. Rev. Lett.* **107**, 136603 (2011).

19. I. Du Lingjie, G. Knez, Sullivan, Du Rui-Rui, Robust helical edge transport in gated InAs/GaSb bilayers. *Phys. Rev. Lett.* **114**, 096802 (2015).

20. K. Bendias, S. Shamim, O. Herrmann, A. Budewitz, P. Shekhar, P. Leubner, J. Kleinlein, E. Bocquillon, H. Buhmann, L. W. Molenkamp, High mobility HgTe microstructures for quantum spin Hall studies. *Nano Lett.* **18**, 4831–4836 (2018).

21. P. Leubner, L. Lunczer, C. Brüne, H. Buhmann, L. W. Molenkamp, Strain engineering of the band gap of HgTe quantum wells using superlattice virtual substrates. *Phys. Rev. Lett.* **117**, 086403 (2016).

22. S. Wiedmann, A. Jost, C. Thienel, C. Brüne, P. Leubner, H. Buhmann, L. W. Molenkamp, J. C. Maan, U. Zeitler, Temperature-driven transition from a semiconductor to a topological insulator. *Phys. Rev. B* **91**, 205311 (2015).

23. S. S. Krishtopenko, I. Yahniuk, D. B. But, V. I. Gavrilenko, W. Knap, F. Teppe, Pressure- and temperature-driven phase transitions in HgTe quantum wells. *Phys. Rev. B* **94**, 245402 (2016).

24. A. M. Kadykov, S. S. Krishtopenko, B. Jouault, W. Desrat, W. Knap, S. Ruffenach, C. Consejo, J. Torres, S. V. Morozov, N. N. Mikhailov, S. A. Dvoretskii, F. Teppe, Temperature-induced topological phase transition in HgTe quantum wells. *Phys. Rev. Lett.* **120**, 086401 (2018).

25. S. S. Krishtopenko, F. Teppe, Quantum spin Hall insulator with a large bandgap, Dirac fermions, and bilayer graphene analog. *Sci. Adv.* **4**, eaap7529 (2018).

26. S. S. Krishtopenko, S. Ruffenach, F. Gonzalez-Posada, G. Boissier, M. Marcinkiewicz, M. A. Fadeev, A. M. Kadykov, V. V. Rumyantsev, S. V. Morozov, V. I. Gavrilenko, C. Consejo, W. Desrat, B. Jouault, W. Knap, E. Tournié, F. Teppe, Temperature-dependent terahertz spectroscopy of inverted-band three-layer InAs/GaSb/InAs quantum well. *Phys. Rev. B* **97**, 245419 (2018).

27. M. Meyer, S. Schmid, F. Jabeen, G. Bastard, F. Hartmann, S. Höfling, Topological band structure in InAs/GaSb/InAs triple quantum wells. *Phys. Rev. B* **104**, 085301 (2021).

28. S. Schmid, M. Meyer, F. Jabeen, G. Bastard, F. Hartmann, S. Höfling, Exploring the phase diagram of InAs/GaSb/InAs trilayer quantum wells. *Phys. Rev. B* **105**, 155304 (2022).

29. E. Tournié, L. M. Bartolome, M. R. Calvo, Z. Loghmari, D. A. Díaz-Thomas, R. Teissier, A. N. Baranov, L. Cerutti, J. B. Rodriguez, Mid-infrared III–V semiconductor lasers epitaxially grown on Si substrates. *Light Sci. Appl.* **11**, 165 (2022).

30. F. Qu, A. J. Beukman, S. Nadj-Perge, M. Wimmer, B. M. Nguyen, W. Yi, J. Thorp, M. Sokolich, A. A. Kiselev, M. J. Manfra, C. M. Marcus, L. P. Kouwenhoven, Electric and magnetic tuning between the trivial and topological phases in InAs/GaSb double quantum wells. *Phys. Rev. Lett.* **115**, 036803 (2015).

31. H. Pan, M. Wu, Y. Liu, S. A. Yang, Electric control of topological phase transitions in Dirac semimetal thin films. *Sci. Rep.* **5**, 14639 (2015).

32. J. L. Collins, A. Tadich, W. Wu, L. C. Gomes, J. N. B. Rodrigues, C. Liu, J. Hellerstedt, H. Ryu, S. Tang, S. K. Mo, S. Adam, S. A. Yang, M. S. Fuhrer, M. T. Edmonds, Electric-field-tuned topological phase transition in ultrathin Na3Bi. *Nature* **564**, 390–394 (2018).

33. M. Karalic, S. Mueller, C. Mittag, K. Pakrouski, Q. Wu, A. A. Soluyanov, M. Troyer, T. Tschirky, W. Wegscheider, K. Ensslin, T. Ihn, Experimental signatures of the inverted phase in InAs/GaSb coupled quantum wells. *Phys. Rev. B* **94**, 241402(R) (2016).

34. M. Meyer, T. Fähndrich, S. Schmid, A. Wolf, S. S. Krishtopenko, B. Jouault, G. Bastard, F. Teppe, F. Hartmann, S. Höfling, Coexistence of topological and normal insulating phases in electro-optically tuned InAs/GaSb bilayer quantum wells. *Phys. Rev. B* **109**, L121303 (2024).

35. D. L. Smith, C. Mailhiot, Proposal for strained type II superlattice infrared detectors. *J. Appl. Phys.* **62**, 2545–2548 (1987).

36. L. Du, T. Li, W. Lou, X. Wu, X. Liu, Z. Han, C. Zhang, G. Sullivan, A. Ikhlassi, K. Chang, R.-R. Du, Tuning edge states in strained-layer InAs/GaInSb quantum spin Hall insulators. *Phys. Rev. Lett.* **119**, 056803 (2017).

37. T. Akiho, F. Couëdo, H. Irie, K. Suzuki, K. Onomitsu, K. Muraki, Engineering quantum spin Hall insulators by strained-layer heterostructures. *Appl. Phys. Lett.* **109**, 192105 (2016).

38. H. Irie, T. Akiho, F. Couëdo, K. Suzuki, K. Onomitsu, K. Muraki, Energy gap tuning and gate-controlled topological phase transition in In As/$In_xGa_{1-x}Sb$ composite quantum wells. *Phys. Rev. Mater.* **4**, 104201 (2020).

39. C. Avogadri, S. Gebert, S. S. Krishtopenko, I. Castillo, C. Consejo, S. Ruffenach, C. Roblin, C. Bray, Y. Krupko, S. Juillaguet, S. Contreras, A. Wolf, F. Hartmann, S. Höfling, G. Boissier, J. B. Rodriguez, S. Nanot, E. Tournié, F. Teppe, B. Jouault, Large inverted band gap in strained three-layer InAs/GaInSb quantum wells. *Phys. Rev. Res.* **4**, L042042 (2022).

40. S. Mueller, C. Mittag, T. Tschirky, C. Charpentier, W. Wegscheider, K. Ensslin, T. Ihn, Edge transport in InAs and InAs/GaSb quantum wells. *Phys. Rev. B* **96**, 075406 (2017).

41. S. Mueller, A. N. Pal, M. Karalic, T. Tschirky, C. Charpentier, W. Wegscheider, K. Ensslin, T. Ihn, Nonlocal transport via edge states in InAs/GaSb coupled quantum wells. *Phys. Rev. B* **92**, 081303(R) (2015).

42. F. Nichele, H. J. Suominen, M. Kjaergaard, C. M. Marcus, E. Sajadi, J. A. Folk, F. Qu, A. J. A. Beukman, F. K. D. Vries, J. van Veen, S. Nadj-Perge, L. P. Kouwenhoven, B. M. Nguyen, A. A. Kiselev, W. Yi, M. Sokolich, M. J. Manfra, E. M. Spanton, K. A. Moler, Edge transport in the trivial phase of InAs/GaSb. *New J. Phys.* **18**, 083005 (2016).

43. M. Brahlek, N. Koirala, N. Bansal, S. Oh, Transport properties of topological insulators: Band bending, bulk metal-to-insulator transition, and weak anti-localization. *Solid State Commun.* **215-216**, 54–62 (2015).

44. C. Fuchs, S. Shamim, P. Shekhar, L. Fürst, J. Kleinlein, J. I. Väyrynen, H. Buhmann, L. W. Molenkamp, Kondo interaction of quantum spin Hall edge channels with charge puddles. *Phys. Rev. B* **108**, 205302 (2023).

45. E. B. Olshanetsky, G. M. Gusev, A. D. Levin, Z. D. Kvon, N. N. Mikhailov, Multifractal conductance fluctuations of helical edge states. *Phys. Rev. Lett.* **131**, 076301 (2023).

46. S. Benlenqwanssa, S. S. Krishtopenko, M. Meyer, B. Benhamou-Bui, L. Bonnet, A. Wolf, C. Bray, C. Consejo, S. Ruffenach, S. Nanot, J.-B. Rodriguez, E. Tournié, F. Hartmann, S.

Höfling, F. Teppe, B. Jouault, Multi-probe analysis to separate edge currents from bulk currents in quantum spin Hall insulators and to analyze their temperature dependence. *Phys. Rev. Appl.* **22**, 064059 (2024).

47. L. Lunczer, P. Leubner, M. Endres, V. L. Müller, C. Brüne, H. Buhmann, L. W. Molenkamp, Approaching quantization in macroscopic quantum spin Hall devices through gate training. *Phys. Rev. Lett.* **123**, 047701 (2019).

48. T. Dietl, Effects of charge dopants in quantum spin Hall materials. *Phys. Rev. Lett.* **130**, 086202 (2023).

49. J. I. Väyrynen, M. Goldstein, L. I. Glazman, Helical edge resistance introduced by charge puddles. *Phys. Rev. Lett.* **110**, 216402 (2013).

50. M. Meyer, S. Schmid, F. Jabeen, G. Bastard, F. Hartmann, S. Höfling, Voltage control of the quantum scattering time in InAs/GaSb/InAs trilayer quantum wells. *New J. Phys.* **25**, 023035 (2023).

51. M. Patrashin, K. Akahane, N. Sekine, I. Hosako, Molecular beam epitaxy of strained-layer InAs/GaInSb superlattices for long-wavelength photodetectors. *J. Cryst. Growth* **477**, 86–90 (2017).

52. I. Vurgaftman, J. R. Meyer, L. R. Ram-Mohan, Band parameters for III-V compound semiconductors and their alloys. *J. Appl. Phys.* **89**, 5815–5875 (2001).

53. M. Büttiker, Four-terminal phase-coherent conductance. *Phys. Rev. Lett.* **57**, 1761–1764 (1986).

54. A. Chandola, R. Pino, P. S. Dutta, Below bandgap optical absorption in tellurium-doped GaSb. *Semicond. Sci. Technol.* **20**, 886–893 (2005).

55. N. Segercrantz, J. Slotte, I. Makkonen, J. Kujala, F. Tuomisto, Y. Song, S. Wang, Point defect balance in epitaxial GaSb. *Appl. Phys. Lett.* **105**, 082113 (2014).

56. J. Buckeridge, T. D. Veal, C. R. A. Catlow, D. O. Scanlon, Intrinsic point defects and the n- and p-type dopability of the narrow gap semiconductors GaSb and InSb. *Phys. Rev. B* **100**, 035207 (2019).

57. E. I. Vaughan, S. Addamane, D. M. Shima, G. Balakrishnan, A. A. Hecht, High-resistivity semi-insulating AlSb on GaAs substrates grown by molecular beam epitaxy. *J. Electron. Mater.* **45**, 2025–2030 (2016).

58. B. Chatterjee, S. A. Ringel, R. Sieg, R. Hoffman, I. Weinberg, Hydrogen passivation of dislocations in InP on GaAs heterostructures. *Appl. Phys. Lett.* **65**, 58–60 (1994).

59. J. S. Liu, M. B. Clavel, R. Pandey, S. Datta, M. Meeker, G. A. Khodaparast, M. K. Hudait, Growth and characterization of metamorphic InAs/GaSb tunnel heterojunction on GaAs by molecular beam epitaxy. *J. Appl. Phys.* **119**, 244308 (2016).

60. C. Cervera, J. B. Rodriguez, J. P. Perez, H. At-Kaci, R. Chaghi, L. Konczewicz, S. Contreras, P. Christol, Unambiguous determination of carrier concentration and mobility for InAs/GaSb superlattice photodiode optimization. *J. Appl. Phys.* **106**, 033709 (2009).

61. S. B. Zhang, Y. Y. Zhang, S. Q. Shen, Robustness of quantum spin Hall effect in an external magnetic field. *Phys. Rev. B* **90**, 115305 (2014).

62. C. A. Li, S. B. Zhang, S. Q. Shen, Hidden edge Dirac point and robust quantum edge transport in InAs/GaSb quantum wells. *Phys. Rev. B* **97**, 045420 (2018).

63. S. S. Krishtopenko, Higher-order topological insulator in cubic semiconductor quantum wells. *Sci. Rep.* **11**, 21060 (2021).
